# Artificial intelligence-driven study selection in systematic reviews of randomized controlled trials, emulated trials and economic evaluation studies using large language models

**DOI:** 10.1371/journal.pdig.0001668

**Published:** 2026-08-28

**Authors:** Panu Looareesuwan, Wanchana Ponthongmak, Amarit Tansawet, Cholatid Ratanatharathorn, Habib ur Rehman Owasi, Gareth J. McKay, John Attia, Takehiko Oami, Ammarin Thakkinstian

**Affiliations:** 1 Department of Clinical Epidemiology and Biostatistics, Faculty of Medicine Ramathibodi Hospital, Mahidol University, Bangkok, Thailand; 2 Faculty of Medicine, Research Affairs, Chulalongkorn University, Bangkok, Thailand; 3 Centre for Public Health, Queen’s University Belfast, Belfast, United Kingdom; 4 Centre for Clinical Epidemiology and Biostatistics, School of Medicine and Public Health, University of Newcastle, Newcastle, NSW, Australia; 5 Department of Emergency and Critical Care Medicine, Chiba University Graduate School of Medicine, Chiba, Japan; Mayo Clinic Arizona, UNITED STATES OF AMERICA

## Abstract

Systematic reviews (SRs) are key to evidence-based medicine but are often labor-intensive, especially in the study selection step. This study assessed the use of large language models (LLMs) to automate SR study screening and selection. Five SR projects were included: two published therapeutic SRs (SR1–2), two ongoing emulated-trial SRs (SR3–4), and one economic evaluation SR (SR5). The total number of studies screened for each SR was 3,966, 3,147, 695, 3,096 and 485, respectively, with 20, 24, 46, 32 and 70 eligible studies. Three LLMs—Gemini 2.0 Flash, Llama 3.1, and Qwen 2.5—were evaluated using training sets (five studies), title/abstract datasets, and full-text datasets predicted as relevant. Prompts based on the PICOS framework were iteratively refined using a recall-first strategy. Outputs were compared with human reviewer classifications using recall, number needed to screen (NNS), and percentage reduced workload with 95% confidence intervals. In the title/abstract screening phase, Llama 3.1 and Gemini 2.0 Flash achieved consistently high recall (90.00%-100.00% and 90.48%-100.00%), with workload reduction of 61.92%-97.10% and 65.21%-97.03%, respectively. Qwen 2.5 achieved the highest workload reduction (76.16%-99.19%) but showed the lowest recall (76.67%-88.89%). In the full-text selection phase, Llama 3.1 achieved the highest recall (93.33%-100.00%) with workload reductions of 73.15%-97.41%, but slower processing time (approximately 2.3-3.6 minutes per document). Qwen 2.5 yielded lower recall (66.67%-89.71%), despite the highest workload reduction (80.82%-99.44%) and similarly slow inference times (approximately 3.0-4.2 minutes per document). Gemini 2.0 Flash balanced high recall (83.33%-100.00%) with substantial workload reduction (76.71%-98.91%) and markedly faster inference (approximately 4–8 seconds per document). LLMs—particularly Llama 3.1 and Gemini 2.0 Flash—can substantially reduce SR screening workload while maintaining high recall when guided by a recall-first prompting framework. Remaining challenges include reproducibility in closed-source models and generalizability across diverse SR topics.

## Author summary

Systematic reviews serve as the cornerstone of evidence-based medicine, providing highly reliable health information by synthesizing all existing research on a specific clinical topic. However, conducting these reviews is a notoriously time-consuming and labor-intensive process. Researchers must manually screen thousands of scientific articles to identify the small fraction of studies that meet strict eligibility criteria. In this study, we explored whether artificial intelligence, specifically large language models, could safely automate this screening workload. We evaluated three different models across five diverse systematic review projects, assessing their ability to read both short abstracts and full-text articles to accurately identify relevant studies. We found that two of these models were highly effective, successfully capturing nearly all eligible studies while reducing the manual human screening workload by up to 99%. Notably, one model processed and evaluated complex full-text articles in a matter of seconds per document. Our findings suggest that large language models can act as highly capable assistants for research teams. By automating the most resource-intensive phases of evidence synthesis, these tools can substantially accelerate the translation of medical research into clinical guidelines, ultimately benefiting public health.

## 1. Introduction

Systematic reviews (SRs) with meta-analyses are important in evidence-based practice. [1] as per their rigorous methodology in synthesizing existing studies [2,3]. However, conducting an SR is notably labor-intensive and time-consuming, particularly during the study screening phase, i.e., screening a large volume of records to identify only relevant studies. This imposes a substantial burden on human reviewers [4] in both effort and time.

Artificial intelligence (AI)-powered tools leveraging natural language processing (NLP) have been developed to support the screening process, e.g., Abstrackr [5], DistillerSR [6], EPPI-Reviewer [7], Rayyan [8], and Covidence [9]. Most existing AI tools [5–9] employ NLP-based supervised machine learning (ML) approaches, which require a substantial number of manually annotated studies to train models for study screening and selection. A key limitation of supervised ML approaches is the uncertainty surrounding the number of annotated studies required for training to achieve acceptable performance. For SRs with a limited number of eligible studies, this uncertainty may require reviewers to annotate most or nearly all identified records during the training phase. As a result, these tools may paradoxically increase rather than reduce reviewer workload, particularly in small-scale reviews.

To address these limitations, few-shot learning (FSL), a meta-learning-based supervised ML approach, has been introduced as a more annotation-efficient solution [10]. In this classifier sense, FSL involves training discriminative, non-generative models (e.g., BERT-based classifiers) to learn similarity patterns between eligible and ineligible studies labeled in the support set. While FSL-based approaches have shown promise in reducing screening workload [11], they still require a minimum number of annotated studies to initiate model training, typically 4–6 eligible and 40–60 ineligible studies. Furthermore, these models are constrained by input length limitations (e.g., 384 tokens), restricting their applicability to only the title/abstract screening phase. Consequently, full-text selection, where richer context information is available, remains largely infeasible using FSL-based classifiers. Moreover, reported workload reductions vary across SR types, highlighting the need for more flexible and scalable solutions.

The emergence of large language models (LLMs) such as ChatGPT (OpenAI) [12], Llama (Meta AI) [13], and Gemini (Google) [14], has opened new possibilities for automating the study screening process in SR workflows. These models, trained on massive corpora with advanced reasoning capability techniques, have recently been explored for use in study screening. Prior studies by Oami et al. [15,16], Guo et al. [17], and Issaiy et al. [18] applied LLMs guided by PICOS-based prompts [population (P), intervention (I), comparator (C), outcome (O), and study design (S)]. While specificity was high (76%-99%), recall varied widely (25%-100%), raising concerns about false negatives (FNs). Although Oami et al. [19] used GPT-3.5 Turbo [20] and GPT-4 Turbo [21] to reduce workload, they only screened titles/abstracts, which is often insufficient for making a decision regarding study inclusion. Unlike traditional methods or FSL, LLMs can process longer and more complex input data, making them more suitable for full-text selection.

Therefore, this study applied both closed- and open-source LLMs to the SR study selection process across both the title/abstract screening and full-text selection phases. Notably, the eligible studies missed by the LLM predictions (FNs) during the title/abstract phase were intentionally included in the input pool for the full-text selection phase. We designed it this way to independently assess accuracy at each stage, because our goal is to use the LLMs in a real-world SR process as a screening tool to support second reviewers and help reduce their workload, rather than as a fully automated end-to-end pipeline. Findings may help researchers in selecting appropriate LLM tools tailored to their settings.

## 2. Materials and methods

### 2.1. Data sources

This study utilized data from five SR projects conducted by the Department of Clinical Epidemiology and Biostatistics, Faculty of Medicine Ramathibodi Hospital, Mahidol University. These comprised two published therapeutic SRs, two ongoing SRs of emulated trials using real-world data, and one economic evaluation study.

Two types of input datasets were extracted from individual studies: 1) Titles and abstracts, 2) Full-text articles. SR1 examined mesh-augmented fascia closure techniques for hernia prophylaxis [22]. Of the 3,966 identified studies, 3,942 were available for screening (24 were excluded due to missing/inaccessible full-text), and 20 were deemed eligible.

SR2 addressed antiviral therapies for cytomegalovirus prophylaxis in kidney transplantation [23]. A total of 3,060 out of 3,147 records (87 missing/inaccessible full-texts) were available for screening, with 23 eligible (one was excluded due to missing full-text).

SR3 evaluated the effectiveness of treatments for osteoporotic fracture prevention using real-world data [24]. Of the 695 identified studies, 577 were included for screening after excluding 118 studies with missing or inaccessible full texts, and 46 studies met the eligibility criteria.

SR4 assessed the effectiveness of high-intensity statins versus moderate-intensity statins using real-world data [25]. Of the 3,096 identified studies, 2,895 were available for screening after excluding 201 studies due to unavailable full texts; 32 studies met the eligibility criteria.

SR5 examined the cost-utility of sodium-glucose cotransporter-2 inhibitors, angiotensin receptor-neprilysin inhibitors, and guideline-directed medical therapy in patients with heart failure [26]. Of the 485 initially identified studies, 370 remained for screening after excluding 115 studies with inaccessible full texts, and 70 studies were included in the final analysis. Median word counts are detailed in Table 1.

**Table 1:** Model systematic review input data characteristics for title/abstract screening.

SR (year)	N of databases	N1/N2	N excluded studies	Title/abstract screening		
				Train dataset (total/eligible)	Title/abstract test dataset (total/eligible)	Words/doc Median (IQR)
SR1 (2020) [22]	2	3,966/20	24	5/2	3,937/18	254 (186-301)
SR2 (2021) [23]	4	3,147/24	87	5/2	3,055/21*	244 (177-298)
SR3 (2024) [24]	3	695/46	118	5/2	572/44	307 (272-349)
SR4 (2025) [25]	2	3,096/32	201	5/2	2,890/30	312 (286-348)
SR5 (2026) [26]	3	485/70	115	5/2	365/68	277 (170-336)

doc: document; IQR: interquartile range; N1/N2: number of identified studies/eligible studies; SR: systematic review; *The number of eligible studies was reduced to 21 due to one eligible study that could not be accessed to retrieve the full-text article.

For ascertainment of the reference standard (ground truth) for study selection, eligibility ascertainment in SR1, SR2, and SR5 was conducted independently by two human reviewers, with disagreements resolved through discussion and adjudication by the third reviewer or review team. For the ongoing projects (SR3 and SR4), initial study selections were conducted by a single reviewer, and the final selections were subsequently validated by senior supervisors with experience in SR methodology.

### 2.2. Dataset

Three datasets were used: 1) Training dataset. Principal investigators of each SR manually selected five representative studies (two eligible and three ineligible) to iteratively develop and refine prompt instructions and decision logic. A minimal set of representative eligible and ineligible studies was chosen to simulate an early-stage SR, when only a limited number of labeled studies are typically available; 2) Title/abstract test dataset. After excluding training studies, the title/abstract screening dataset comprised the following: SR1: 3,937 studies (18 eligible); SR2: 3,055 (21 eligible); SR3: 572 (44 eligible); SR4: 2,890 (30 eligible); SR5: 365 (68 eligible). Data characteristics are summarized in Table 1; and 3) Full-text test dataset: Full-text datasets included studies classified during title/abstract screening as true positives (TPs), false positives (FPs), and FNs for each LLM (i.e., Llama 3.1 [27], Qwen 2.5 [28], and Gemini 2.0 Flash [29]). Full-texts were selected to enable evaluation of studies identified by human reviewers (TP + FN) as well as those selected by LLMs (TP + FP). By intentionally including the FNs from the title/abstract screening phase, we assess the LLM’s full-text capability independently. This evaluates how well it performs as a supportive tool for a second reviewer, rather than testing a continuous pipeline where those missed studies would simply be lost.

Full-text articles were retrieved from electronic databases and converted into plain text using PyMuPDF [30] for articles in PDF format and the Nougat model [31] for scanned documents. The size of the full-text test datasets varied for both SR and LLM (see Table 2). In SR1, the full-text test dataset included 117 studies for Gemini 2.0 Flash, 114 for Llama 3.1, and 34 for Qwen 2.5 (all containing 18 eligible studies). For SR2, the corresponding full-text test datasets comprised 316, 346 and 60 studies, respectively (all containing 21 eligible studies). For SR3, Gemini 2.0 Flash, Llama 3.1, and Qwen 2.5 contained 131, 160 and 110 full-text articles (44 eligible studies) for the full-text test datasets. In SR4, the full-text test datasets consisted of 135, 271, and 107 studies (30 eligible studies), and in SR5, 127, 140, and 99 studies were evaluated, respectively (68 eligible studies). The median number of words per document of all full-text test datasets ranged from approximately 5,200–7,300.

**Table 2:** Model systematic review input data characteristics for full-text selection.

SR (year)	LLM	Title/abstract test dataset (total/eligible)	Full-text test dataset (total/eligible)	Words/doc Median (IQR)
SR1 (2020) [22]	Gemini 2.0 Flash	3,937/18	117/18	5,378 (4,340-6,876)
	Llama 3.1		114/18	5,196 (4,168-6,538)
	Qwen 2.5		34/18	5,478 (5,028-6,980)
SR2 (2021) [23]	Gemini 2.0 Flash	3,055/21	316/21	6,159 (4,363-9,243)
	Llama 3.1		346/21	5,724 (3,545-8,634)
	Qwen 2.5		60/21	5,348 (3,289-7,312)
SR3 (2024) [24]	Gemini 2.0 Flash	572/44	131/44	7,296 (5,890-8,798)
	Llama 3.1		160/44	7,199 (5,880-8,825)
	Qwen 2.5		110/44	7,365 (6,010-8,812)
SR4 (2025) [25]	Gemini 2.0 Flash	2,890/30	135/30	6,723 (5,601-7,890)
	Llama 3.1		271/30	6,732 (5,680-8,084)
	Qwen 2.5		107/30	7,098 (5,698-8,442)
SR5 (2026) [26]	Gemini 2.0 Flash	365/68	127/68	6,536 (2,728-7,970)
	Llama 3.1		140/68	7,093 (5,264-8,423)
	Qwen 2.5		99/68	7,093 (5,264-8,350)

doc: document; IQR: interquartile range; LLM: large language model; SR: systematic review.

### 2.3. Model framework

**Fig 1:**
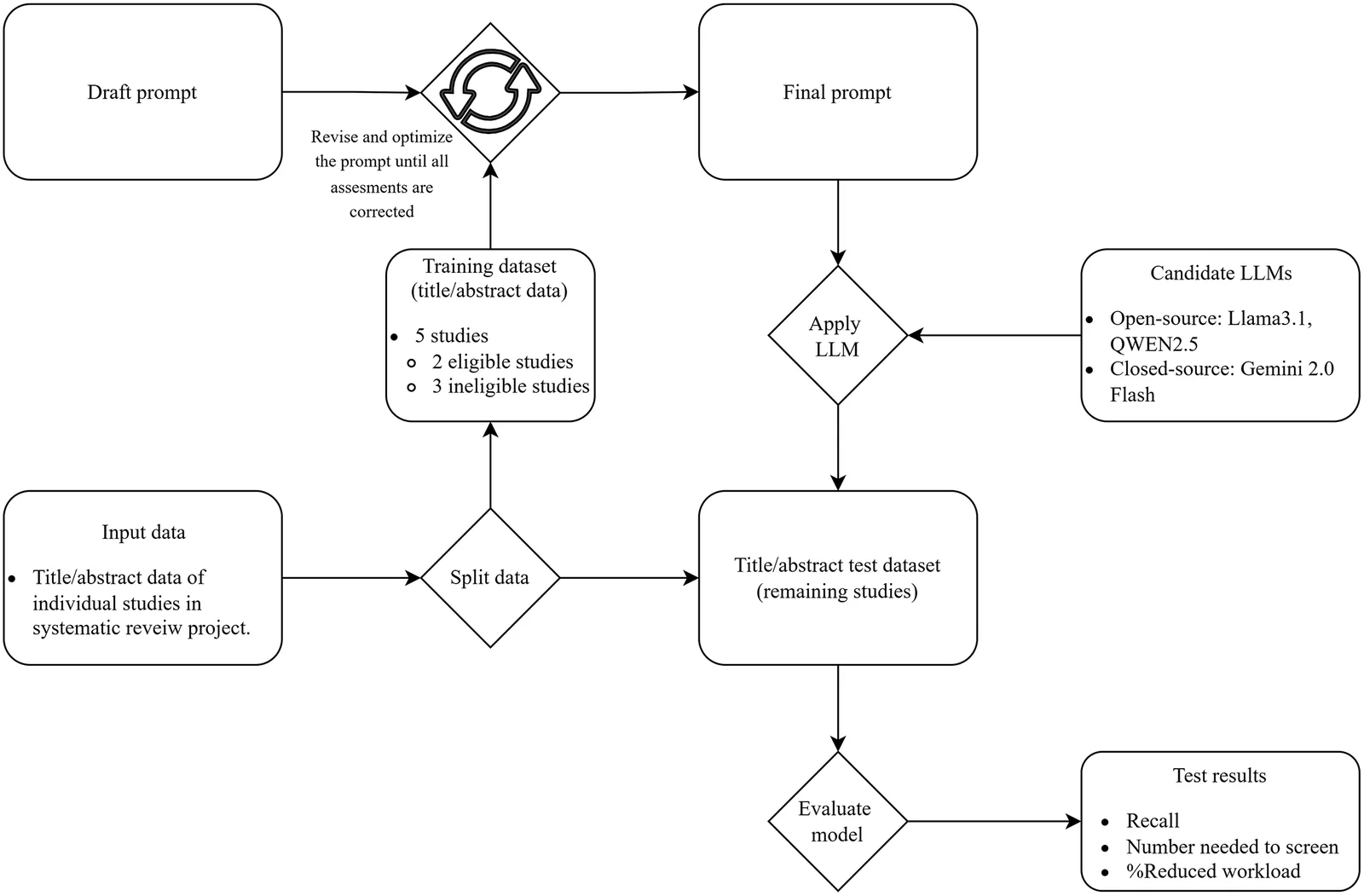
Model framework for the title and abstract screening phase.

**Fig 2:**
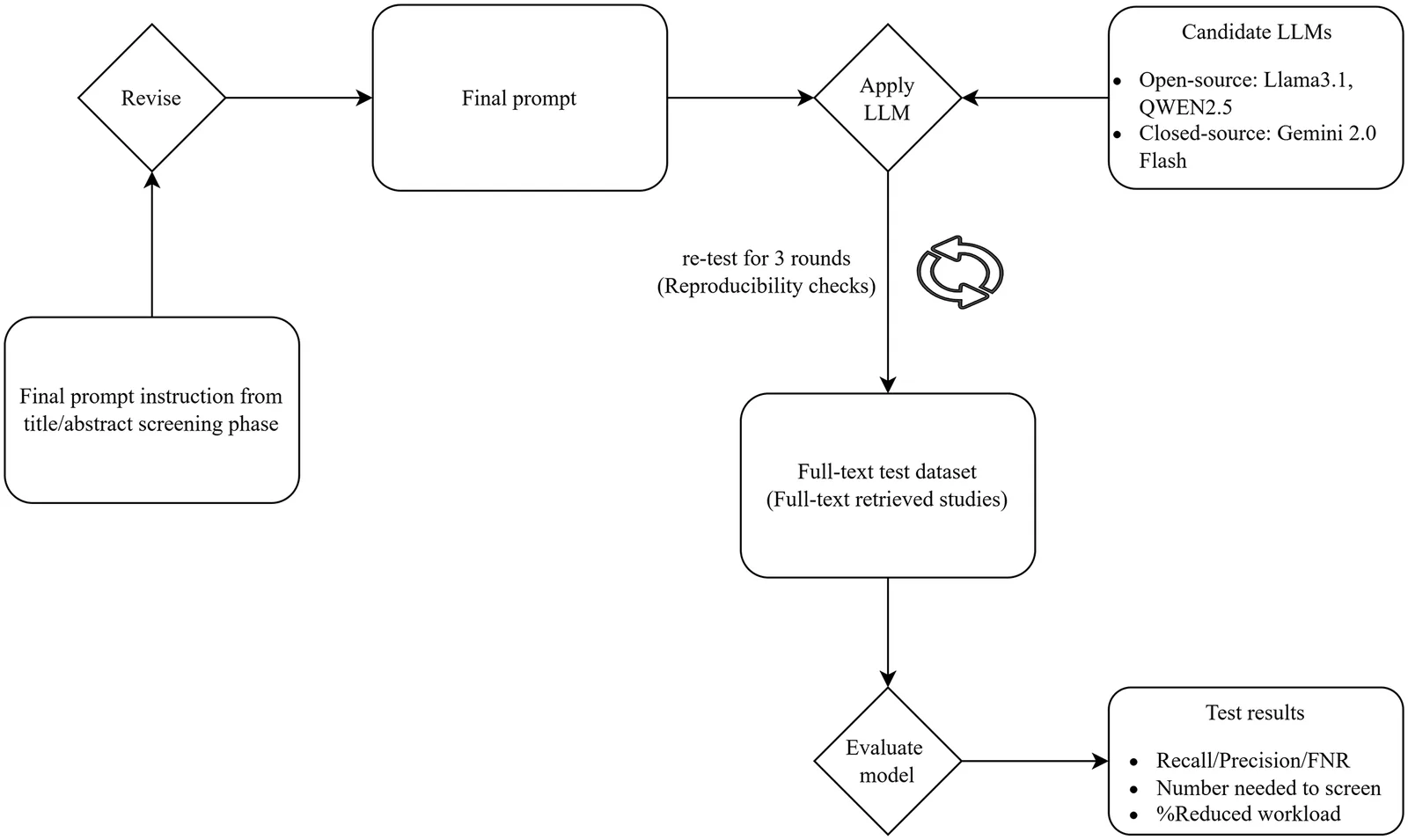
Model framework for the full text selection phase.

Prompts were developed for open-source (Llama 3.1 [27] [released on July 23^rd^, 2024], and Qwen 2.5 [28] [September 19^th^, 2024]) and closed-source (Gemini 1.5 Flash, subsequently upgraded to Gemini 2.0 Flash [29] [February 5^th^, 2025]) multilingual LLMs. During the study period, the closed-source model transitioned from Gemini 1.5 Flash to Gemini 2.0 Flash. To maintain methodological consistency, the same prompt structure, inclusion/exclusion criteria, decision logic, and instructions were applied across both model versions. No modifications to the prompting framework were required following the model upgrade. All LLM-assisted study selections were executed via Python scripts through API integration to ensure reproducibility and scalability. All LLM frameworks were implemented across two phases: title/abstract screening, and full-text selection (see Figs 1 and 2), from October 1^st^, 2024, to January 12^th^, 2026. For the closed-source LLM, Gemini 2.0 Flash supports a maximum context length of 1,048,576 tokens, whereas Llama 3.1 and Qwen 2.5 allow up to 128,000 tokens. In this study, the two open-source LLMs were constrained to 16,384 tokens per input to match the available computational infrastructure.

All open-source LLM runs were conducted on a local workstation equipped with an NVIDIA RTX 4090 GPU, 256 GB of RAM, and an AMD Ryzen 9 9950X 16-core CPU (4.30 GHz), which provided sufficient capacity for long-context processing and reproducible execution.

All evaluated LLMs support multilingual input, and therefore also support the screening and selection frameworks for non-English studies without prior translation. However, because the evaluation datasets consisted mainly of English-language publications, cross-language performance was not directly assessed.

#### 2.3.1. Title/abstract screening phase.

**Fig 3:**
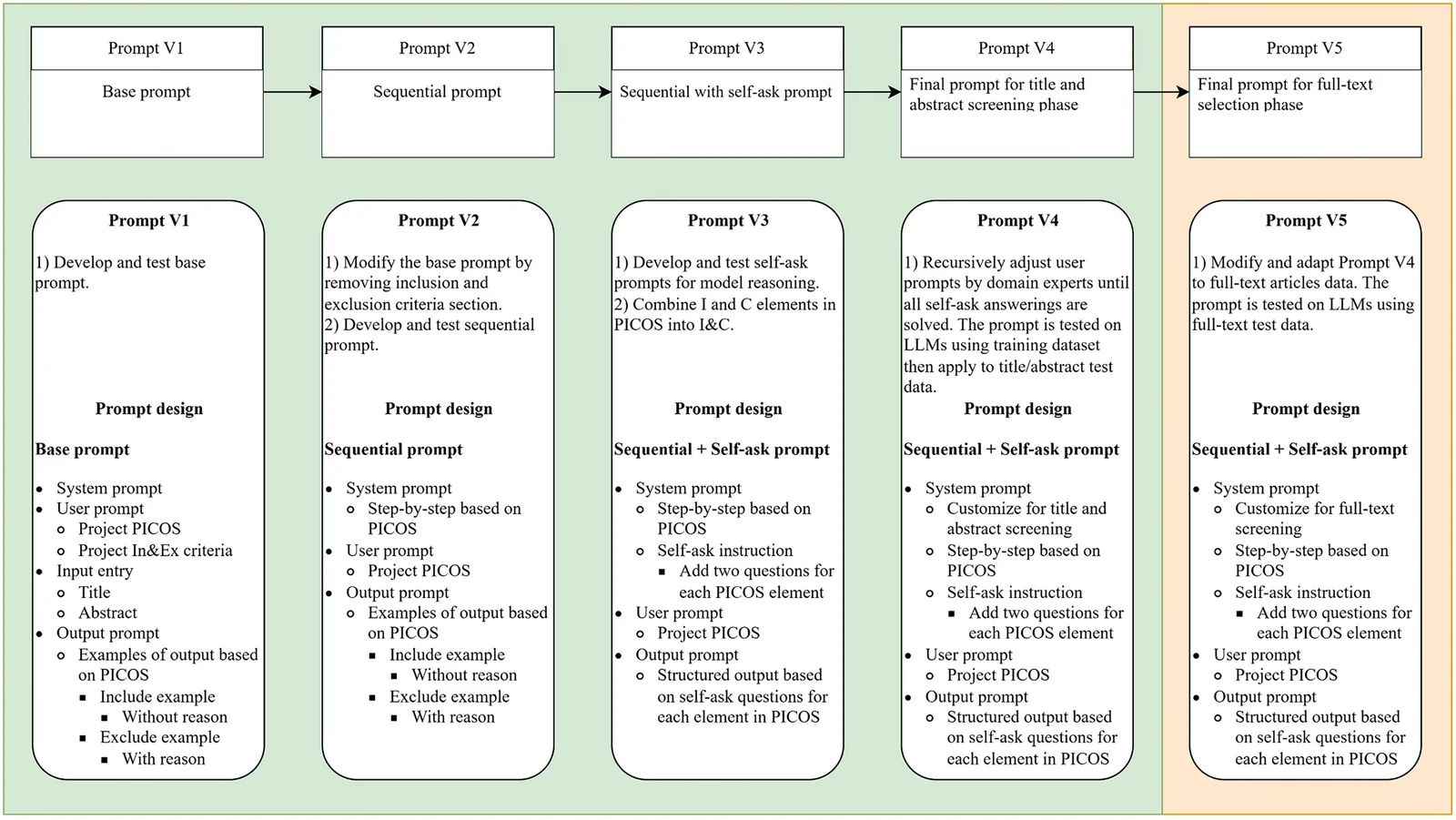
Prompt development stages.

An overview of the model framework is shown in Fig 3. Prompt development followed established prompt engineering guidelines [32]. Initial prompts were developed based on the PICOS framework and iteratively trained and refined using training data (two eligible and three ineligible). Prompt development proceeded through four versions as follows:

1) Prompt V1: Base prompt

Prompt V1 served as the initial structured template and was composed of four core components:

First, the system prompt defined the role of the LLM as an assistant reviewer for SR study selection. The instructions were based on the PICOS framework and the preferred reporting items for systematic reviews and meta-analyses (PRISMA) guidelines [33], ensuring alignment with established standard practices.

Second, the user prompt specified information relevant to the PICOS elements, along with the inclusion and exclusion criteria specific to each SR.

Third, the input section provided a designated space for the input title/abstract of each study, ensuring systematic organization for LLM processing.

Finally, the output prompt defined the expected output format, requiring classification of studies as either eligible or ineligible. For ineligible studies, the LLM was instructed to provide explicit reasons for exclusion based on the PICOS criteria, thereby ensuring transparency and interpretability of the selection process.

2) Prompt V2: Sequential prompt

Prompt V2 retained the four key structural components of Prompt V1 but addressed its primary limitation, i.e., an insufficient explanation of decision logic. To better emulate the conventional study screening and selection process by human reviewers, Prompt V2 implemented a sequential evaluation of the P, I, C, O, and S domains. Specifically, the LLMs first evaluated the P criterion. If satisfied, the LLMs returned a “True” response and proceeded to assess the next domain. If the criterion was not satisfied, the LLM returned a “False” response, stopped further processing, and provided the reason for ineligibility. This sequential evaluation was repeated for the remaining I, C, O, and S domains.

In this round, the predefined inclusion and exclusion criteria were omitted from the user prompt, as these criteria were inherently embedded within the PICOS framework. Re-providing them was deemed redundant and potentially confusing for the LLMs. This modification aimed to promote a more concise, transparent, and structured decision-making process, ensuring clear and reproducible eligibility determinations.

3) Prompt V3: Sequential prompt with self-asking

Prompt V3 was developed to further enhance logical reasoning and decision transparency of the LLMs. Unlike Prompt V2, which required a classification response before justification, Prompt V3 encouraged explicit critical thinking prior to decision-making. By requiring the models to explicitly articulate their reasoning for each PICOS criterion, this approach aimed to ensure that study selection was grounded in a brief self-check, where the model re-asks each PICOS eligibility question to organize its thinking before returning the final “True” or “False” decision.

To simplify implementation, the I and C elements were combined into a single “I&C” domain, as these domains were difficult to separate in some SRs, leaving four domains in the PICOS framework. For each P, I&C, O, and S domain, the LLMs had to answer two intermediate questions: 1) “What content is related to this domain?” and 2) “Does this match with the corresponding P, I&C, O, S criterion?”

The LLMs were required to answer these intermediate questions before providing justification. Similar to Prompt V2, the LLMs initially evaluated the P domain; if the criterion was met, they answered “True”; otherwise, “False”. This process was then repeated for the remaining I&C, O, and S domains. To better reflect real-practice title/abstract screening, where some PICOS information may be incompletely reported in the title/abstract, but may be present in the full-text, the following decision rules were established in the system prompt: (i) prioritize inclusion over exclusion, unless each PICOS criterion is clearly and explicitly not met; (ii) when information is unclear or missing in the title/abstract, the LLMs should assume the criterion might be met in the full-text and respond “True”; (iii) return “False” only when the title/abstract explicitly contradicts a PICOS domain. After evaluating all PICOS elements, the LLMs determined the final classification using a logical decision rule: (i) if all PICOS domains were labeled “True”, the study was classified as eligible (“True”); and (ii) if any of the PICOS domains were labeled “False”, the study was classified as ineligible (“False”).

Structuring the reasoning process in this stepwise manner enhanced transparency, improved classification accuracy, and reduced the risk of misclassification due to insufficient contextual analysis.

4) Prompt V4: Refined prompt

Although Prompt V3 represented the final structural template, further refinements were required within the user prompt to ensure precise contextual alignment with the specific requirements and eligibility criteria of each SR.

To achieve this, the principal reviewers and expert team collaboratively reviewed and discussed the context-specific aspects of the PICOS framework for each SR. Model outputs generated during the initial prompt development phase (V1-V5) and based on the training dataset for the five sample studies were jointly examined with attention to both the eligibility classification and accompanying justifications.

Consequently, the PICOS descriptions and prompt instructions were iteratively refined with the explicit goal of achieving a targeted recall of 100%, regardless of workload reduction or precision. Refinements continued until the LLMs’ eligibility decisions and justifications were deemed acceptable or no further meaningful improvements could be identified. Once finalized, the optimized prompts (see S1-S5 Tables) were applied uniformly to the title/abstract test datasets for formal model evaluation.

#### 2.3.2. Full-text selection phase.

The full-text articles typically contain more comprehensive and detailed information than the title/abstract. Consequently, it was expected that all PICOS domains would be explicitly addressed at this phase. Prompt V5, adapted from V4 in the title/abstract screening phase, incorporated a full-text selection process. It required “True” or “False” responses across all PICOS domains, reflecting standard selection.

The system prompt was updated by including more specific inclusion and/or exclusion criteria. If all PICOS domains were satisfied, the full-text article was marked as “True” (eligible); if any criterion was “False”, missing, ambiguous, or unclear, the final decision was “False” (ineligible). The finalized prompt V5 (see S6-S10 Tables) was applied to the full-text article test dataset to assess the final decision and overall model performance.

### 2.4. Model evaluation

LLM performance was evaluated against human reviewer classifications (reference standard, as detailed in section 2.1 Data sources). The following metrics were used according to a standard confusion matrix for model framework evaluation (Table 3).

**Table 3:** The confusion matrix for model framework evaluation.

LLM	Traditional reviewer		
	Eligible	Ineligible	Total
Eligible	TP	FP	TP + FP
Ineligible	FN	TN	FN + TN
Total	N-eligible	N-ineligible	N

FN: false negatives; FP: false positives; LLM: large language model; N: number of studies; TN: true negatives; TP: true positives;

1) Recall (sensitivity) is the proportion of truly eligible studies correctly identified by the LLMs:

$$\begin{array}{c} Recall=\;\frac{TP}{TP+FN} \end{array}$$

2) Precision is the proportion of studies classified as eligible by the LLM that were truly eligible:

$$\begin{array}{c} Precision=\frac{TP}{TP+FP} \end{array}$$

3) False Negative Rate (FNR) is the proportion of missed eligible studies relative to all actual eligible studies:

$$\begin{array}{c} FNR=\frac{FN}{TP+F\;N} \end{array}$$

4) Number needed to screen (NNS) is the total number of studies classified by the LLM as potentially eligible (NNS_1_ referred to NNS for the title/abstract screening phase, while NNS_2_ was for the full-text selection phase):

$$\begin{array}{c} NNS=TP+FP \end{array}$$

5) Number needed to read (NNR) is the expected number of LLM-identified eligible studies that must be manually reviewed to obtain one truly eligible study:

$$\begin{array}{c} NNR=\;\frac{\text{1}}{Precision} \end{array}$$

6) Percentage of workload reduction represents the reduction in the number of studies needing human review:

$$\begin{array}{c} \%\;Reduced\;workload=\left(\frac{N-(TP+FP)}{N}\right)\times \text{1}\text{0}\text{0} \end{array}$$

7) The 95% confidence interval (CI) for proportions (e.g., recall, precision, and % reduced workload) was estimated using two approaches depending on the observed proportion. For proportions involving boundary values (i.e., 0% or 100%), the Wilson score method was applied because the standard Wald method may yield inaccurate confidence intervals under these conditions. The Wilson-adjusted proportion ($\tilde{p_{i}}$) and standard error (SE) were calculated as:

$$\begin{array}{c} \tilde{p_{i}}=\;\frac{\hat{p_{i}}+\frac{z^{\text{2}}}{\text{2}n_{i}}}{\text{1}+\frac{z^{\text{2}}}{n_{i}}} \end{array}$$

$$\begin{array}{c} {SE}_{Wilson}=\frac{z}{\text{1}+\frac{z^{\text{2}}}{n_{i}}}\sqrt{\frac{{\hat{p}}_{i}\left(\text{1}-{\hat{p}}_{i}\right)}{n_{i}}+\frac{z^{\text{2}}}{{\text{4}n}_{i}^{\text{2}}}} \end{array}$$

The corresponding 95% CI was estimated as:


$$\begin{array}{c} {\text{95}\%\;CI}_{Wilson}=\;\tilde{p_{i}}\pm \frac{z}{\text{1}+\frac{z^{\text{2}}}{n_{i}}}\sqrt{\frac{{\hat{p}}_{i}\left(\text{1}-{\hat{p}}_{i}\right)}{n_{i}}+\frac{z^{\text{2}}}{{\text{4}n}_{i}^{\text{2}}}} \end{array}$$

Where $\hat{p_{i}}$ is the observed proportion, $n_{i}$ is the total number of studies contributing to the estimate, and z is the standard normal critical value (i.e., 1.96 for a two-sided CI).

For proportions that did not involve boundary values (i.e., neither 0% nor 100%), the Wald method was used. The SE and 95% CI were calculated as:


$$\begin{array}{c} \text{9}\text{5}\%\;CI\;\hat{p_{i}}=\hat{p_{i}}\pm Z_{\propto /\text{2}}{SE}_{i} \end{array}$$

$$\begin{array}{c} {SE}_{i}\;=\;\sqrt{\frac{\hat{p_{i}}\left(\text{1}-\hat{p_{i}}\right)}{n_{i}}} \end{array}$$

Comparisons between two proportions were performed using a two-sided Z test:


$$\begin{array}{c} Z\;=\frac{\left({\hat{p}}_{A}-\;{\hat{p}}_{B}\right)}{\sqrt{SE_{A}^{\text{2}}+\;SE_{B}^{\text{2}}}} \end{array}$$

$$\begin{array}{c} P-value\;=\;\text{2}\left(\text{1}\;-\;\Phi \left(|Z|\right)\right) \end{array}$$

Where ${\hat{p}}_{A}$ and ${\hat{p}}_{B}$ are the proportions for model A and model B, obtained using either the Wilson or Wald method as appropriate; SE_A_ and SE_B_ are their respective standard errors; Φ represents the cumulative distribution function of the standard normal distribution.

8) Processing time is the average time required per document by each LLM, used to assess computational efficiency.

In line with standard SR practice, recall was treated as the primary performance criterion, as missing eligible studies (FN) poses a greater risk of bias to the validity of evidence synthesis than reviewing additional ineligible studies. Reviewers, therefore, typically accept higher false-positive rates in exchange for minimizing the risk of excluding eligible studies. Precision, NNS, NNR, and % reduced workload were used to quantify the manual workload associated with achieving high recall.

Metrics were computed separately for each LLM across both phases, enabling comparison of each model’s ability to maintain high recall while minimizing manual workload. Test-retest reliability was evaluated by executing each LLM independently three times under identical prompt conditions. For Llama 3.1 and Qwen 2.5, random seeds were fixed across runs to enable reproducibility. Consistency of final classifications was then evaluated across repeated executions.

### 2.5. Error analysis

Error analysis examined discordant cases between LLM and human selections from both title/abstract screening and full-text selection phases. Key transitions analyzed included “FN→TP”, “TP→FN”, and “FN→FN” (incorrectly classified by both stages). Reasoning in LLM-generated outputs for each PICOS domain was reviewed. Misclassified cases were manually assessed to identify error patterns and evaluate LLM consistency and adaptability across both selection phases.

## 3. Results

### 3.1. Title/abstract screening phase

LLM confusion matrices for title/abstract screening and performance metrics are presented in Table 4 and Table 5, respectively, with pairwise comparison tests summarized in S11 Table.

**Table 4:** Confusion matrix for large language models title and abstract screening phase.

SR (year)	LLM	N studies	TP	FP	FN	TN	Time/doc
SR1 (2020) [22]	Gemini	3,937	18	99	0	3,820	4s
	Llama	3,937	18	96	0	3,823	1m 8s
	Qwen	3,937	16	16	2	3,903	56s
SR2 (2021) [23]	Gemini	3,055	19	295	2	2,739	4s
	Llama	3,055	20	325	1	2,709	1m 59s
	Qwen	3,055	17	39	4	2,995	1m 46s
SR3 (2024) [24]	Gemini	572	44	87	0	441	4s
	Llama	572	44	116	0	412	1m 9s
	Qwen	572	36	66	8	462	54s
SR4 (2025) [25]	Gemini	2,890	30	105	0	2,755	4s
	Llama	2,890	27	241	3	2,619	1m 9s
	Qwen	2,890	23	77	7	2,783	53s
SR5 (2026) [26]	Gemini	365	68	59	0	238	5s
	Llama	365	67	72	1	238	1m 23s
	Qwen	365	56	31	12	225	59s

doc, document; FN, false negatives; FP, false positives; LLM, large language model; m, minutes; s, seconds; SR, systematic review; Time/doc, average time per document; TN, true negatives; TP, true positives.

**Table 5:** Performance of large language models in the title and abstract screening phase.

SR	LLM	%Precision (95% CI)	%Recall (95% CI)	%FNR (95% CI)	%RW (95% CI)	NNS_1_	NNR
SR1 (2020) [22]	Gemini 2.0	15.38 (8.84-21.92)	100.00 (77.29-100.00)	0.00 (0.00-22.71)	97.03 (96.50-97.56)	117	6.50
	Llama 3.1	15.79 (9.10-22.48)	100.00 (77.29-100.00)	0.00 (0.00-22.71)	97.10 (96.58-97.62)	114	6.33
	Qwen 2.5	50.00 (32.68-67.32)	88.89 (74.37-100.00)	11.11 (0.00-25.63)	99.19 (98.91-99.47)	32	2.00
SR2 (2021) [23]	Gemini 2.0	6.05 (3.41-8.69)	90.48 (77.93-100.00)	9.52 (0.00-22.07)	89.72 (88.64-90.80)	314	16.53
	Llama 3.1	5.80 (3.33-8.27)	95.24 (86.13-100.00)	4.76 (0.00-13.87)	88.71 (87.59-89.83)	345	17.24
	Qwen 2.5	30.36 (18.32-42.40)	80.95 (64.15-97.75)	19.05 (2.25-35.85)	98.17 (97.69-98.65)	56	3.29
SR3 (2024) [24]	Gemini 2.0	33.59 (25.50-41.68)	100.00 (89.31-100.00)	0.00 (0.00-10.69)	77.10 (73.66-80.54)	131	2.98
	Llama 3.1	27.50 (20.58-34.42)	100.00 (89.31-100.00)	0.00 (0.00-10.69)	72.03 (68.35-75.71)	160	3.64
	Qwen 2.5	35.29 (26.02-44.56)	81.82 (70.42-93.22)	18.18 (6.78-29.58)	82.17 (79.03-85.31)	102	2.83
SR4 (2025) [25]	Gemini 2.0	22.22 (15.21-29.23)	100.00 (85.05-100.00)	0.00 (0.00-14.95)	95.33 (94.56-96.10)	135	4.50
	Llama 3.1	10.07 (6.47-13.67)	90.00 (79.26-100.00)	10.00 (0.00-20.74)	90.73 (89.67-91.79)	268	9.93
	Qwen 2.5	23.00 (14.75-31.25)	76.67 (61.54-91.80)	23.33 (8.20-38.46)	96.54 (95.87-97.21)	100	4.35
SR5 (2026) [26]	Gemini 2.0	53.54 (44.87-62.21)	100.00 (92.82-100.00)	0.00 (0.00-7.18)	65.21 (60.32-70.10)	127	1.87
	Llama 3.1	48.20 (39.89-56.51)	98.53 (95.67-100.00)	1.47 (0.00-4.33)	61.92 (56.94-66.90)	139	2.07
	Qwen 2.5	64.37 (54.31-74.43)	82.35 (73.29-91.41)	17.65 (8.59-26.71)	76.16 (71.79-80.53)	87	1.55

FNR, false negative rate; LLM, large language model; NNR, number needed to read; NNS_1_, number needed to screen in title/abstract screening phase; SR, systematic review; %RW, % reduced workload; 95% CI, 95% confidence interval.

Note: Wald 95% CIs are used by default. Wilson 95% CIs are used only for perfect evaluation scores, so boundary estimates retain finite-sample uncertainty.

Across all SRs, Gemini 2.0 Flash and Llama 3.1 consistently achieved very high recall (often reaching 100.00%), whereas Qwen 2.5 achieved greater workload reduction at the expense of lower recall.

In SR1, both Gemini 2.0 Flash and Llama 3.1 achieved perfect recall (100.00% [95% CI: 77.29%-100.00%]), while Qwen 2.5 showed a lower recall of 88.89% (95% CI: 74.37%-100.00%); however, these recalls were not significantly different (all p-values >0.05). Qwen 2.5 achieved the highest % reduced workload (99.19% [95% CI: 98.91%-99.47%], NNS₁ = 32), which was significantly greater than that of Gemini 2.0 Flash (97.03% [95% CI: 96.50%-97.56%], p-value <0.001, NNS₁ = 117) and Llama 3.1 (97.10% [95% CI: 96.58%-97.63%], p-value <0.001, NNS₁ = 114). No significant difference was observed between Gemini 2.0 Flash and Llama 3.1 (p-value = 0.854). These findings indicate that although Qwen 2.5 achieved approximately 2% additional workload reduction, this efficiency gain was at the cost of a higher rate of missed eligible studies (FNR = 11.11% [95% CI: 0.00%-25.63%]). In contrast, Gemini 2.0 Flash and Llama 3.1 preserved perfect recall (FNR = 0.00% [95% CI: 0.00%-22.71%]), prioritizing recall over maximal workload reduction.

For SR2, Llama 3.1 achieved the highest recall at 95.24% (95% CI: 86.13%-100.00%), followed by Gemini 2.0 Flash at 90.48% (95% CI: 77.93%-100.00%) and Qwen 2.5 at 80.95% (95% CI: 64.15%-97.75%). Differences in recall across these LLMs were not statistically significant (all p-values >0.05). Qwen 2.5 achieved the highest workload reduction at 98.17% (95% CI: 97.69%-98.65%, NNS₁ = 56), which was significantly greater than Gemini 2.0 Flash (89.72% [95% CI: 88.64%-90.80%], p-value <0.001, NNS₁ = 314) and Llama 3.1 (88.71% [95% CI: 87.58%-89.83%], p-value <0.001, NNS₁ = 345). The difference in workload reduction between Gemini 2.0 Flash and Llama 3.1 was not statistically significant (p-values = 0.203). Consistent with SR1, the higher workload reduction achieved by Qwen 2.5 was accompanied by a markedly higher FNR (19.05% [95% CI: 2.25%-35.85%]), compared with Gemini 2.0 Flash (9.52% [95% CI: 0.00%-22.07%]) and Llama 3.1 (4.76% [95% CI: 0.00%-13.87%]).

In SR3, Gemini 2.0 Flash and Llama 3.1 both achieved perfect recall (100.00% [95% CI: 89.31%-100.00%]), whereas Qwen 2.5 showed lower recall (81.82% [95% CI: 70.42%-93.22%]), though these differences did not reach statistical significance (p-value = 0.110 for both pairwise comparisons). Workload reductions differed significantly across the three LLMs. Qwen 2.5 achieved the highest workload reduction at 82.17% (95% CI: 79.03%-85.31%), followed by Gemini 2.0 Flash at 77.10% ([73.65%-80.54%], p-value = 0.033), and Llama 3.1 at 72.03% ([68.35%-75.71%], p-value <0.001). However, the higher workload reduction achieved by Qwen 2.5 was accompanied by the highest FNR at 18.18% (95% CI: 6.78%-29.58%), whereas Gemini 2.0 Flash and Llama 3.1 maintained FNRs of 0.00% (0.00%-10.69%), reflecting perfect recall but substantially lower workload reduction relative to Qwen 2.5.

In SR4, Gemini 2.0 Flash again achieved perfect recall (100.00% [95% CI: 85.05%-100.00%]), followed by Llama 3.1 (90.00% [95% CI: 79.26%-100.00%]), and Qwen 2.5 (76.67% [95% CI: 61.54%-91.80%]); the differences between these models did not reach statistical significance (p-value >0.05 for both pairwise comparisons). Workload reduction was highest in Qwen 2.5 (96.54% [95% CI: 95.87%-97.21%]), which was significantly higher than Gemini 2.0 Flash (95.33% [95% CI: 94.56%-96.10%], p-value = 0.020), and Llama 3.1 (90.73% [95% CI: 89.67%-91.79%], p-value <0.001). This additional workload reduction achieved by Qwen 2.5 was accompanied by a higher FNR (23.33% [95% CI: 8.20%-38.46%]), whereas Gemini 2.0 Flash and Llama 3.1 showed lower FNRs.

In SR5, Qwen 2.5 demonstrated significantly lower recall (82.35% [95% CI: 73.29%-91.41%]) compared with Gemini 2.0 Flash (100.00% [95% CI: 92.82%-100.00%], p-value = 0.020) and Llama 3.1 (98.53% [95% CI: 95.67%-100.00%], p-value <0.001), while recall did not differ significantly between Gemini 2.0 Flash and Llama 3.1 (p-value = 0.799). Qwen 2.5 again achieved the greatest workload reduction but exhibited the highest FNR at 17.65% (95% CI: 8.59%-26.71%), compared with 0.00% (95% CI: 0.00%-7.18%) for Gemini 2.0 Flash and 1.47% (95% CI: 0.00%-4.33%) for Llama 3.1.

Overall, Gemini 2.0 Flash demonstrated the most favorable balance between recall and workload reduction, achieving a median (range) recall of 100.00% (90.48%-100.00%), a median NNS₁ of 131 (117–314), and a median % reduced workload of 89.72% (65.21%-97.03%). Llama 3.1 achieved similarly high recall with a median of 98.53% [90.00%-100.00%] but required a higher screening effort (median NNS₁ 160 [114–345]), resulting in slightly lower workload reduction (median: 88.71% [61.92%-97.10%]). In contrast, Qwen 2.5 consistently minimized screening burden, with the lowest median NNS₁ of 87 (32–102), and the highest median workload reduction (96.54% [76.16%-99.19%]); however, these gains came at the cost of substantially lower recall (median: 81.82% [76.67%-88.89%]) and higher FNR.

Despite its strong overall performance, Llama 3.1 occasionally produced less stringent reasoning than Gemini 2.0 Flash. For example, in a study of midline incisional hernias conducted in rodents, Llama 3.1 marked the P domain as “True”, reasoning that “*Although the study uses a rodent model, it could potentially provide insights relevant to humans undergoing similar procedures*”.

In contrast, Gemini 2.0 Flash correctly labeled this study as “False”, with reasoning that “*The study is conducted on rodents, not humans, which does not align with the population criteria*”. Although Qwen 2.5 also misclassified this study as “True”, its reasoning appropriately acknowledged “*Although the study uses a rodent model, the description of midline incisions aligns with the population criteria. Further evaluation of the full text would be necessary to confirm if the study can be extrapolated to human subjects*”. These qualitative differences highlight meaningful variation in reasoning rigor across LLMs, even when headline performance metrics appear comparable.

### 3.2. Full-text selection phase

**Fig 4:**
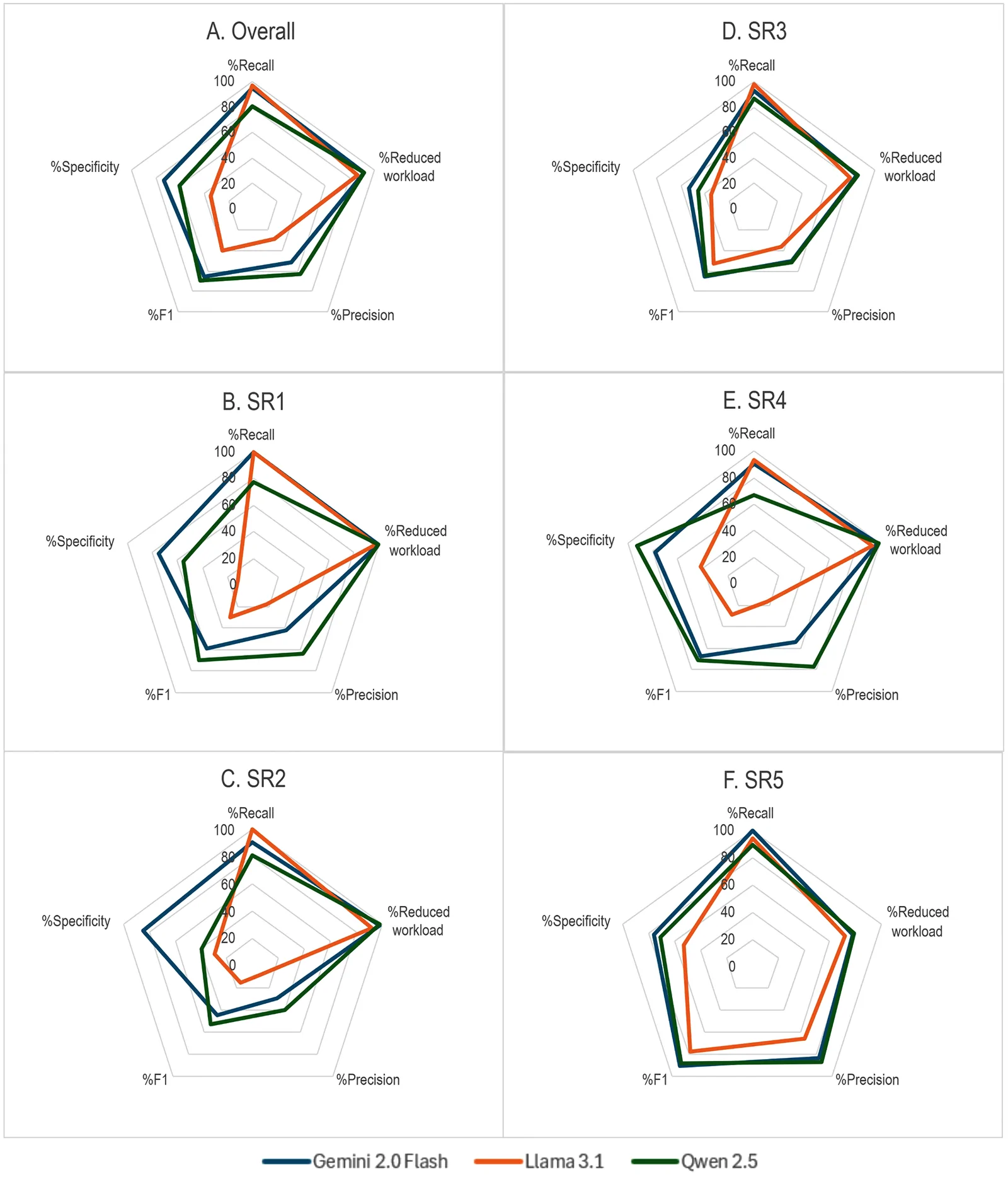
Comparative performance of large language models in the full-text selection phase across five systematic reviews.

In this phase, the input data for each LLM comprised studies classified as TP, FP, and FN during the title/abstract screening phase. Consequently, the number and composition of studies evaluated at this phase differed across LLMs, reflecting their respective workload reductions achieved during title/abstract screening. Based on model-specific full-text datasets, the resulting confusion matrices and derived performance metrics are summarized in S12 Table and S13 Table. The overall performance of the three LLMs across all five full-text test datasets is summarized in Fig 4A.

For SR1, recall and % reduced workload performance of the three LLMs are illustrated in Fig 4B. Both Gemini 2.0 Flash and Llama 3.1 achieved perfect recall (100.00% [95% CI: 77.29%-100.00%]) across all three rounds, which was higher than Qwen 2.5 (77.78% [95% CI: 58.57%-96.99%]), though these differences did not reach statistical significance (p-value = 0.430 for both comparisons). No significant difference in recall was observed between Gemini 2.0 Flash and Llama 3.1 (p-value = 1.000). Gemini 2.0 Flash required screening of 43–44 studies (NNS_2_), corresponding to a workload reduction of 98.88%-98.91% (95% CI: 98.55%-99.23%), whereas Llama 3.1 required screening of 102 studies, resulting in a workload reduction of 97.41% (95% CI: 96.91%-97.91%). In contrast, Qwen 2.5 required only 22 NNS_2_ studies, resulting in the highest % reduced workload of 99.44% (95% CI: 99.21%-99.67%). Qwen 2.5's workload reduction was significantly greater than that of Gemini 2.0 Flash (p-value = 0.009) and Llama 3.1 (p-value <0.001), and Gemini 2.0 Flash was significantly greater than Llama 3.1 (p-value <0.001). In terms of processing time per document, Gemini 2.0 Flash was the fastest (~6 seconds/document), followed by Llama 3.1 (~2 minutes 14–29 seconds/document) and Qwen 2.5 (~3 minutes 32 seconds/document).

Performance metrics for the three LLMs for SR2 are illustrated in Fig 4C. Llama 3.1 again achieved perfect recall (100.00% [95% CI: 79.90%-100.00%], FN = 0) but had the highest NNS_2_, i.e., it required screening of 250 studies, resulting in a % reduced workload of 91.82% ([95% CI: 90.85%-92.79%]). Gemini 2.0 Flash demonstrated a better balance between recall and % reduced workload, with slightly lower recall (90.48% [95% CI: 77.93%-100.00%]) but substantially lower NNS_2_ of 64–65 studies and a higher workload reduction of 97.87%-97.91% ([95% CI: 97.36%-98.42%]). Qwen 2.5 exhibited the lowest recall (80.95% [95% CI: 64.15%-97.75%]) but achieved the highest workload reduction (98.59% [95% CI: 98.17%-99.01%]), requiring screening of only 43 studies. There were no significant differences in recall between the three models (all p-values >0.05). However, the % reduced workload of Qwen 2.5 was significantly greater than Gemini 2.0 Flash (p-value = 0.043) and Llama 3.1 (p-value <0.001), and Gemini 2.0 Flash was significantly greater than Llama 3.1 (p-value <0.001). Consequently, Qwen 2.5 yielded the highest FN rate (19.05% [95% CI: 2.25%-35.85%]), followed by Gemini 2.0 Flash (9.52% [95% CI: 0.00%-22.07%]), whereas Llama 3.1 had no FNs (0.00% [95% CI: 0.00%-20.10%]). In terms of efficiency, Gemini 2.0 Flash remained the fastest model, processing each document in approximately 7–8 seconds, whereas both Llama 3.1 and Qwen 2.5 required approximately 3 minutes per document.

For SR3, Gemini 2.0 Flash achieved high recall ranging from 88.64% to 93.18% ([95% CI: 79.26%-100.00%]) while requiring only 78–82 studies to screen (NNS_2_), corresponding to a workload reduction of 85.66%-86.36% ([95% CI: 82.79%-89.17%]), see Fig 4D. Llama 3.1 showed the highest recall (97.73% [95% CI: 93.33%-100.00%]) but required substantially more studies to be screened (NNS_2_ = 118), resulting in a lower workload reduction of 79.37% ([95% CI: 76.05%-82.69%]). Qwen 2.5 achieved the lowest recall (86.36% [95% CI: 76.22%-96.50%]) but required screening of only 74 studies, yielding a workload reduction of 87.06% ([95% CI: 84.31%-89.81%]). While Llama 3.1 had significantly higher recall than Qwen 2.5 (p-value = 0.044), no significant differences were observed between Gemini 2.0 Flash and the other two models (p-values >0.05). For workload reduction, Qwen 2.5 and Gemini 2.0 Flash showed no significant difference (p-value = 0.490), but both were significantly greater than Llama 3.1 (p-values <0.001 and = 0.005, respectively). Consequently, Qwen 2.5 yielded the highest FN rate (13.64% [95% CI: 3.50%-23.78%]), followed by Gemini 2.0 Flash (6.82%-11.36% [95% CI: 0.00%-20.74%]), whereas Llama 3.1 had the lowest FN rate (2.27% [95% CI: 0.00%-6.67%]). Gemini 2.0 Flash again showed the best efficiency, requiring approximately 5–6 seconds/document, whereas Llama 3.1 and Qwen 2.5 spent approximately 3 minutes 31–34 seconds/document.

For SR4, Gemini 2.0 Flash achieved a recall ranging from 83.33% to 90.00% ([95% CI: 69.99%-100.00%]) requiring screening of 47–50 studies (NNS_2_), corresponding to a % reduced workload of 98.27%-98.37% ([95% CI: 97.79%-98.83%]), see Fig 4E. Llama 3.1 achieved the highest recall (93.33% [95% CI: 84.40%-100.00%]) but required more screening (168 studies), leading to a lower workload reduction of 94.19% ([95% CI: 93.34%-95.04%]). Qwen 2.5 showed the lowest recall (66.67% [95% CI: 49.80%-83.54%]) but achieved the highest workload reduction (99.10% [95% CI:98.76%-99.44%]) and required screening of only 26 studies. Both Gemini 2.0 Flash and Llama 3.1 achieved significantly higher recall than Qwen 2.5 (p-values = 0.022 and 0.006, respectively), with no significant difference between them (p-value = 0.640). Qwen 2.5 workload reduction was significantly greater than Gemini 2.0 Flash (p-value = 0.006) and Llama 3.1 (p-value <0.001), while Gemini 2.0 Flash was significantly greater than Llama 3.1 (p-value <0.001). Consequently, Qwen 2.5 yielded the highest FN rate (33.33% [95% CI: 16.46%-50.20%]), followed by Gemini 2.0 Flash (10.00%-16.67% [95% CI: 0.00%-30.01%]), whereas Llama 3.1 had the lowest FN rate (6.67% [95% CI: 0.00%-15.60%]). Gemini 2.0 Flash processed documents substantially faster (~5 seconds/document) than Llama 3.1 (~3 minutes 1 second) and Qwen 2.5 (~4 minutes 11 seconds).

For SR5, Gemini 2.0 Flash achieved perfect recall (100.00% [95% CI: 92.82%-100.00%]) across all three rounds, requiring 82–85 studies for NNS_2_ and yielding a workload reduction of 76.71%-77.53% ([95% CI: 72.37%-81.81%]), see Fig 4F. Llama 3.1 showed slightly lower recall (94.12% [95% CI: 88.53%-99.71%]), and required screening of more studies (NNS_2_ = 98), resulting in a % reduced workload of 73.15% ([95% CI: 68.60%-77.70%]). Qwen 2.5 achieved a recall of 89.71% ([95% CI: 82.49%-96.93%]) while requiring screening of only 70 studies, producing a workload reduction of 80.82% ([95% CI: 76.78%-84.86%]). No significant differences in recall were observed between the three models (all p-values >0.05). For workload reduction, Qwen 2.5 was significantly greater than Llama 3.1 (p-value = 0.013), but no significant differences were observed between Gemini 2.0 Flash and the other two models (all p-values >0.05). Consequently, Qwen 2.5 yielded the highest FN rate (10.29% [95% CI: 3.07%-17.51%]), followed by Llama 3.1 (5.88% [95% CI: 0.29%-11.47%]), whereas Gemini 2.0 Flash had no FNs (0.00% [95% CI: 0.00%-7.18%]). Gemini 2.0 Flash required approximately 4–5 seconds/document, whereas Llama 3.1 and Qwen 2.5 required approximately 2–4 minutes/document.

Overall, Llama 3.1 yielded the best protection against FNs, ranging from 0% to 6.67%, but at the cost of lower workload reduction (ranging from 73.15% in SR5 to 97.41% in SR1) and **s**ubstantially slower processing time (approximately 2.3-3.6 minutes/document), Fig 4A. In contrast, Qwen 2.5 maximized workload reduction from 80.82% up to 99.44% but incurred substantially higher FN rates (10.29%-33.33%), and similarly slow inference times (approximately 3.0-4.2 minutes/document). Gemini 2.0 Flash had the most consistently balanced near-perfect recall (FN = 0.00%-16.67%) with a substantial workload reduction (76.71%-98.91%), while also providing markedly faster inference, completing screening in approximately 4–8 seconds per document.

### 3.3. Reproducibility analysis

To assess reproducibility, all LLMs were evaluated over three repeated runs. Open-source models (i.e., Llama 3.1 and Qwen 2.5) allowed for seed control, enabling consistent, reproducible outputs. In contrast, the Gemini model did not support seed setting, resulting in slight performance variation across runs. For instance, in SR1, the number needed to screen in the full-text selection phase (NNS_2_) for Gemini 2.0 Flash varied slightly (43–44 studies), while recall remained constant at 100.00% across all three runs. Similar modest fluctuations in NNS_2_ were observed for Gemini 2.0 Flash in other full-text test datasets (i.e., 64–65 in SR2, 78–82 in SR3, 47–50 in SR4, and 82–85 in SR5). This same pattern of minor variability was observed across all performance metrics.

Across all five SRs, Gemini 2.0 Flash consistently maintained high recall and closely similar workload reduction values across repeated runs, indicating stable performance despite the lack of seed control. Similarly, both Llama 3.1 and Qwen 2.5 produced identical predictions and performance metrics across all three runs for each SR, reflecting perfect reproducibility under fixed random seeds (S12 Table). Overall, these findings indicate that all three LLMs demonstrated high reproducibility in the full-text selection phase, with only minimal variability observed for Gemini 2.0 Flash.

### 3.4. Error analysis

Error analysis of FNs in the title/abstract screening and full-text selection is summarized in S14 Table. Llama 3.1 demonstrated perfect recall in SR1 and SR2 (FN = 0.00%) and nearly perfect recall in SR3-SR5 (93.33%-97.73%), corresponding to FN rates of 0%-6.67%. Gemini 2.0 Flash achieved perfect recall in SR1 and SR5, and near-perfect in SR2-SR4 (83.33%-93.18%), with FNs of 0.00%-16.67%. In contrast, Qwen 2.5 showed consistently lower recall (66.67% -89.71%) and higher FN rates (10.29%-33.33%).

For Gemini 2.0 Flash, most FNs were attributable to difficulties interpreting the P-domain, particularly when patient eligibility was implicitly defined in the original included studies. Common errors included failure to recognize majority-based eligibility (e.g., ≥ 50% meeting P criteria), misclassification of age subgroups (e.g., > 40 years or >70 years) that were valid subsets of broader adult patients (≥18 years), and inability to infer patients’ eligibility indirectly (e.g., patients receiving anti-osteoporotic medications without an explicit diagnosis).

For Llama 3.1, FNs primarily resulted from misinterpretation of complex I&C structures. Errors frequently occurred when the comparators were not stated clearly or were indirectly described, embedded in a multi-arm design, or labeled as “standard care”. Additional errors arose from failure to identify emulated trials in the S domain, including studies using propensity score analyses or target trial frameworks.

For Qwen 2.5, most FNs were mainly driven by misinterpretation of both I&C and O domains. A frequent error involved incorrect handling of “any-of the following” logic, whereby the model required all listed criteria to be met rather than at least one. Further errors reflected limited clinical domain knowledge, such as failure to recognize percutaneous coronary intervention as a valid intervention for patients with atherosclerotic cardiovascular disease, or to identify guideline-directed medical therapies in heart failure patients.

Phase-to-phase consistency between title/abstract screening and full-text selection was examined using all possible classification transitions, see S15 Table. Gemini 2.0 Flash and Llama 3.1 showed high stability, retaining all TPs (TP → TP) in SR1 and SR5 for Gemini 2.0 Flash; SR1 and SR2 for Llama 3.1, and exhibiting limited TP → FN transitions (ranging from 1 to 5 and from 1 to 3, respectively). Both LLMs also reclassified large numbers of FPs as TNs (FP → TN) during the full-text selection phase, contributing to workload reduction while preserving high recall.

In contrast, Qwen 2.5 exhibited lower phase-to-phase stability, with TP → FN transitions observed in all five SRs (ranging from 1 to 8), leading to consistent recall loss despite improvements in specificity (FP → TN) across SRs. Overall, these transition patterns indicate greater reliability and consistency for Gemini 2.0 Flash and Llama 3.1 compared with systematic phase-to-phase instability in Qwen 2.5.

## 4. Discussion

### 4.1. Main findings

This study evaluated the performance, efficiency, and reproducibility of LLMs for automating the study screening of the title/abstract and selection of the full-text phases, across five SRs (two therapies, two emulated trials, and one economic evaluation). We found that both Gemini 2.0 Flash and Llama 3.1 consistently demonstrated the highest recall, achieving either perfect or near-perfect recall across both screening and study selection phases. This finding is particularly important in the context of evidence synthesis, where achieving very high recall, and consequently very few FNs (missed eligible studies) is methodologically critical. Exclusion of eligible studies may introduce selection bias and distort the evidence base, whereas higher FP rates primarily increase the manual screening workload.

Gemini 2.0 Flash exhibited substantially faster processing speed and greater workload reduction, whereas Llama 3.1 demonstrated comparable recall with higher test-retest reproducibility enabled by the ability to fix random seeds. Despite this similar performance, both models demonstrate important practical trade-off considerations. In practice, Gemini 2.0 Flash offers faster inference and more consistent reasoning quality, making it well-suited for large-scale SR or time-sensitive screening tasks. In contrast, Llama 3.1 provides greater transparency, which may be preferable in settings prioritizing auditability and methodological controls, but this comes at the cost of slower processing speed and less robust reasoning in some cases. Additional considerations include deployment and cost: Gemini 2.0 Flash requires paid API access and operates within a closed-source infrastructure, whereas Llama 3.1 can be deployed locally without per-inference costs, provided sufficient computational resources are available. These findings underscore that model choice should be guided not only by accuracy metrics but also by operational constraints and study priorities, including speed, cost, transparency, and reproducibility.

Our prompt refinement strategy explicitly prioritized achieving a targeted recall of 100.00%, irrespective of workload reduction or precision. Consequently, although Llama 3.1 and Gemini 2.0 Flash exhibited excellent recalls, their precision values were substantially lower (5.80%-48.20% and 6.05%-53.54%, respectively, during the title/abstract screening across SR1-SR5). In practice, this implies that reviewers would still need to manually screen a large number of FP studies. Consistent with the prioritization of recall over precision in evidence synthesis, such a trade-off is often acceptable because FPs primarily increase manual review workload, whereas FNs risk permanently omitting eligible relevant studies and creating bias within the evidence base. From a practical perspective, Gemini 2.0 Flash and Llama 3.1 may therefore be most suitable as high-recall filtering tools to support second-reviewer screening or to operate in parallel with human reviewers, rather than as a fully automated tool to replace human judgment. In contrast, Qwen 2.5 offered high workload reduction but at the cost of substantially lower recall, a trade-off that is generally unacceptable in SR contexts.

The observed misclassification patterns mapped closely to three main limitations associated with LLMs: conceptual knowledge failures, logical failures, and evidence identification failures. Conceptual knowledge failures occurred when models failed to recognize clinically equivalent concepts or domain relationships, indicating limitations in semantic generalization and domain understanding. Logical failures arose when LLMs misunderstood eligibility logic or prompt instructions, such as age threshold criteria, “any-of” logic, or comparator definitions, highlighting weaknesses in rule composition and multi-step reasoning. Evidence identification failures occurred when the LLMs had difficulty identifying key eligibility information from long or dense documents. Together, these patterns indicate that most misclassifications were not driven by missing information alone but instead reflected fundamental limitations in semantic interpretation, rule-based reasoning, and information extraction.

We demonstrate that LLM frameworks can be applied depending on the status of the SR review process, but regardless of which framework is used, the principal reviewer needs to provide several eligible and ineligible studies to facilitate LLM training. First, assuming that principal reviewers have already completed their selections, our LLM frameworks are aimed at reducing the burden on second reviewers. A total NNS_1_ selected by the LLM in the screening title/abstract phase (i.e., TPs and FPs) plus the FN numbers, if any, can be used for further manual review by the second reviewer; alternatively, LLMs can be used for full-text selections depending on the infrastructure and capability in preparing full-text data. Final results should be compared to the principal reviewers’ selections, which serve as the ground truth. Second, if the principal reviewer has not yet completed the selection of studies, LLMs can be used as the second reviewer in parallel. However, this purpose requires the whole title/abstract and full-text data input. Finally, a confusion matrix compare the LLM and traditional reviewer outputs should be constructed; discordant pairs (FPs and FNs) should be explored by the SR team.

### 4.2. Comparison with previous studies

Several real-world ML tools have been developed to support semi-automated SR screening. Among these, Abstrackr has been the most extensively evaluated, with reported workload reductions ranging from 10% to 88% and recall rates between 79% and 97% across multiple studies [34]. Further results were reported with 15%-43% workload reduction and FNRs between 0% and 14% [35], while another evaluation of Abstrackr found workload reductions of 9%-80% with FNRs between 2% and 21% [36]. Another comparative study has shown median workload reductions of approximately 40%, 49%, and 35% for Abstrackr, DistillerSR, and RobotAnalyst, respectively [37]. Under a strict constraint of 100% recall, Tsou et al. [38] reported workload reductions of 4%-49% for Abstrackr and 9%-60% for EPPI-Reviewer.

To improve generalization and screening efficiency under minimal labeling conditions, meta-learning and FSL frameworks were subsequently introduced into SR automation. Wiwatthanasetthakarn et al. [11] reported that FSL-based approaches achieved workload reductions ranging from 51% to 98%, along with FNRs ranging from 2% to 12%, substantially exceeding those reported for traditional SR automation tools.

More recently, LLMs have been explored as an alternative paradigm for SR automation. Prior studies [17–19] demonstrated that LLMs could replicate human screening decisions using title/abstract inputs, but with highly variable recall ranging from 25% to 100%. Consistent with these results, we observed that while the workload reduction was generally high, recall performance varied depending on the LLM, SR topic, and screening phase.

In the title/abstract phase, Gemini 2.0 Flash achieved a median (range) workload reduction of 89.72% (65.21%-97.03%) with a median FNR of 0.00% (0.00%-9.52%), whereas Llama 3.1 yielded a similarly low median FNR of 1.47% (0.00%-10.00%) with slightly lower workload reduction (median: 88.71% [61.92%-97.10%]).

In contrast to both traditional ML tools and earlier LLM-based studies, our study shows that LLM-based screening can achieve workload-recall trade-offs that match or exceed the best previously reported results, while extending automation beyond title/abstract screening to the full-text phase. In the title/abstract phase, Gemini 2.0 Flash achieved a median (range) workload reduction of 89.72% (65.21%-97.03%) with a median recall of 100.00% (90.48%-100.00%) and a median FNR of 0.00% (0.00%-9.52%), exceeding the recall reported for most traditional tools and comparable to the strongest FSL-based approaches. Llama 3.1 likewise achieved a high median recall of 98.53% (90.00%-100.00%) with a low median FNR of 1.47% (0.00%-10.00%) and a slightly lower workload reduction (median: 88.71% [61.92%-97.10%]). These results indicate that LLMs can simultaneously preserve near-perfect recall and achieve workload reductions comparable to the best previously reported SR automation frameworks, while requiring no task-specific model training and supporting both abstract-level and full-text selection.

### 4.3. Strengths and limitations

Our study has a number of strengths. First, prompts were systematically structured, iteratively developed, and extensively refined by an expert team and were properly tailored to the context of each SR. The LLMs were used to emulate real-world review workflows, including both screening title/abstract and full-text selections. High recalls were achieved by Llama 3.1 and Gemini 2.0 Flash, especially during the full-text selection phase, where eligibility decisions require more complex domain-specific reasoning.

Second, this study uniquely introduced an evaluation of reproducibility, highlighting important differences between open-source and closed-source LLMs, an aspect largely unaddressed in prior work. These findings provide practical insights into the trade-offs between transparency and operational efficiency. Specifically, closed-source LLMs (e.g., Gemini 2.0 Flash) offered superior speed but may introduce variability across runs due to the lack of seed control, whereas open-source LLMs (e.g., Llama 3.1) allowed fixed seeds and reproducible outputs with greater transparency.

Third, the inclusion of diverse SR types (two completed therapeutic SRs, two ongoing emulated trial SRs, and one published economic evaluation) suggests that the LLM-based screening framework may be adaptable beyond just therapeutic agent SRs. Evaluation of ongoing SRs also suggested that LLMs may function effectively as a second reviewer, and that discrepancies should be resolved through senior human adjudication.

Finally, the evaluated LLMs demonstrated the ability to handle multilingual studies without requiring prior translation. Across SR1-SR5, three non-English papers were eligible. Gemini 2.0 Flash and Llama 3.1 needed to read 32 and 21 non-English full-texts, respectively, and both achieved perfect recall (100.00%). However, Gemini 2.0 Flash had a much lower FP rate (6.69%) than Llama 3.1 (55.56%), indicating a substantially lower screening burden. In contrast, Qwen 2.5 screened only three non-English full-text papers with one TP and two FNs. Overall, Gemini 2.0 Flash showed the best balance of recall and efficiency for non-English full-texts, while Llama 3.1 traded efficiency for recall, and Qwen 2.5 performed poorly.

However, several limitations must be acknowledged. First, although the reference standard for study selection was established using experienced human reviewers, the procedures were not similar across all SRs. In SR3 and SR4, study selection was initially performed by single reviewer and subsequently validated by senior supervisors rather than through independent duplicate review. Consequently, some disagreement between LLMs and the reference standard may reflect variability in human judgment and interpretation of eligibility criteria rather than true model errors. Nevertheless, we conducted detailed error-analyses of disagreements between LLMs and human decisions, to further assess whether discrepancies were attributable to LLM misclassification or potential inconsistencies among human reviewers.

Second, reproducibility was evaluated using only three repeated runs and for only a single closed-source LLM (Gemini 2.0 Flash), providing a limited assessment of run-to-run variability. Open-source LLMs were assessed using a single hardware configuration. Further studies should evaluate reproducibility across a broader range of runs, different hardware environments, and alternative API endpoints.

Third, although open-source LLMs such as Llama 3.1 demonstrated high recall, their reasoning quality was sometimes poorer and less consistent, likely reflecting constraints on model size and local computational resources with moderate local hardware, which necessarily limited the use of larger models. In contrast, closed-source LLMs are typically hosted on high-performance computers, which may enhance their ability to integrate complex eligibility criteria.

Fourth, an ethical limitation arises from reliance on automated study selection. LLM outputs may be potentially biased through embedded pre-training data or prompt development, possibly leading to the exclusion of eligible important studies. In the context of SR, such omissions are ethically relevant because they may distort and bias the evidence base and influence downstream syntheses and conclusions. This underscores the importance of maintaining human oversight and using LLMs as decision-support tools rather than replacements for human reviewers.

### 4.4. Future directions

Our LLM-based framework can be amended for ease of application to SR. Further studies should prospectively assess the performance of different LLMs across different workflows, including their use as second reviewers in either title/abstract screening or full-text selection, or both phases in parallel. In addition, future research should aim to evaluate performance in a broader range of SR types, including diagnostic, risk, prognostic, and genetic studies, as well as multilingual publications.

Hybrid strategies that combine the complementary strengths of multiple LLMs also merit further prospective investigation. For example, a two-stage screening pipeline could use a high-recall model such as Llama 3.1 for conservative screening in the title/abstract phase, followed by a faster or stronger-reasoning model such as Gemini 2.0 Flash to refine selections in the full-text phase. This approach may offer valuable pathways for improving performance while optimizing computational cost and reviewer workload.

Mitigating misclassification errors, particularly TP → FN transitions, is critical, as these missed eligible studies directly increase the risk of bias in evidence synthesis. Prompt designs should therefore be iteratively refined during pilot experiments in the training dataset development to achieve near or perfect recall, even at the expense of reduced workload efficiency. This process requires a systematic understanding of model-specific behaviours and can be supported by incorporating few-shot examples, explicitly defining eligibility and exclusion criteria, adding structured reasoning instructions, and integrating domain-specific knowledge (e.g., clinical synonym tables) to reduce reasoning failures in borderline or complex cases.

Moreover, there is a critical need to develop standardized evaluation frameworks and guidelines that address reproducibility, reasoning transparency, and user-prompt optimization. Establishing such standards will be essential to ensure the safe, reliable, and scalable adoption of LLMs for evidence synthesis in diverse research domains. Finally, we are developing a web-based application that integrates these LLM-based screening frameworks into a user-friendly interface for SR reviewers. This platform will enable users to upload screening datasets, configure hybrid model pipelines, inspect model decisions, and export prioritized record lists for human review, with the goal of reducing screening workload while maintaining high recall and methodological rigor.

## 5. Conclusions

This study demonstrated the strong potential of open-source Llama 3.1 and closed-source Gemini 2.0 Flash for automating both title/abstract screening and full-text selection. These LLMs achieved high recall, substantial workload reduction, and acceptable reproducibility when supported by optimized prompting frameworks. The framework may help reduce reviewer workload and support the study selection process by enabling LLMs to function as decision-support tools under human supervision. Further refinements to prompt design, evaluation across diverse SR domains, prospective testing of hybrid multi-model screening strategies, development of standardized frameworks for reproducibility and reasoning transparency, and implementation of user-facing tools represent critical next steps toward the reliable and scalable integration of LLM-based screening into SR workflows.

## Supporting information

S1 Table The final prompt for the title and abstract screening phase for systematic review 1. (DOCX)

S2 Table The final prompt for the title and abstract screening phase for systematic review 2. (DOCX)

S3 Table The final prompt for the title and abstract screening phase for systematic review 3. (DOCX)

S4 Table The final prompt for the title and abstract screening phase for systematic review 4. (DOCX)

S5 Table The final prompt for the title and abstract screening phase for systematic review 5. (DOCX)

S6 Table The final prompt for the full-text selection phase for systematic review 1. (DOCX)

S7 Table The final prompt for the full-text selection phase for systematic review 2. (DOCX)

S8 Table The final prompt for the full-text selection phase for systematic review 3. (DOCX)

S9 Table The final prompt for the full-text selection phase for systematic review 4. (DOCX)

S10 Table The final prompt for the full-text selection phase for systematic review 5. (DOCX)

S11 Table Performance and pairwise comparison of large language models in the title and abstract screening phase. (DOCX)

S12 Table The confusion and performance metrics of large language models in the full-text selection phase. (DOCX)

S13 Table Performance and pairwise comparison of large language models in the full-text selection phase. (DOCX)

S14 Table Error analysis based on false negatives in the title and abstract screening phase and final classification in the full-text selection phase of each large language model. (DOCX)

S15 Table The prediction results from the title and abstract screening to the full-text selection phases. (DOCX)
