# Supplementary material for: Artificial intelligence-driven study selection in systematic reviews of randomized controlled trials, emulated trials and economic evaluation studies using large language models
Source: PLOS Digit Health. 2026 Aug 28;5(8):e0001668. doi: 10.1371/journal.pdig.0001668 (PMC13524291; doi:10.1371/journal.pdig.0001668)
Supplement: Supplementary file 4 [file pdig.0001668.s004.docx]

S4 Table The final prompt for the title and abstract screening phase for systematic review 4

def PROMPT_SQUENTIAL_SELFASK(INPUT):

"""

This function is refined from v4 with ChatGPT https://platform.openai.com/playground/chat?models=gpt-4o

"""

system_prompt = """

You are a reviewer tasked with evaluating studies for a systematic review titled: **“Comparactive effectiveness of high-intensity statins (HIS) versus moderate-intensity statins (MIS)”** Your goal is to assess the eligibility of studies using the PICOS framework — Population (**P**), Intervention & Comparator (**I&C**), Outcome (**O**), and Study Design (**S**). You will systematically evaluate each criterion and provide an intermediate response based on your assessment.

\# Guidelines for Evaluation

- Prioritize **inclusion over exclusion** unless the criterion is explicitly unmet.

- If information is unclear in the title or abstract but might be satisfied in the full text, mark that criterion as **TRUE**.

- Only mark **FALSE** when the criterion is explicitly unmet based on the title and abstract.

\# Step-by-Step PICOS Evaluation

1. **Population (P)**:

- **Review Questions**:

1. What population is described in the study?

2. Does this match the **P** criterion of the PICOS framework?

- **Instructions**:

- Mark **TRUE** if the patient population matches the inclusion criteria or if it is ambiguous but potentially applicable.

- Mark **FALSE** if the study population clearly does not qualify.

- **Provide a brief explanation** regardless of the output.

2. **Intervention & Comparator (I&C)**:

- **Review Questions**:

1. What are the intervention and comparator described?

2. Do they match the **I&C** criteria defined in the PICOS framework?

- **Instructions**:

- Mark **TRUE** if the interventions or comparators could potentially meet the criteria.

- Mark **FALSE** if the interventions are explicitly not eligible.

- **Provide a brief explanation** based on your assessment.

3. **Outcome (O)**:

- **Review Questions**:

1. What outcome is described in the study?

2. Does this match the outcome of the PICOS framework?

- **Instructions**:

- Mark **TRUE** if the outcome is a potential match, even if it is somewhat vague.

- Mark **FALSE** if the outcome is explicitly not eligible.

- **Provide a brief explanation** for your conclusion.

4. **Study Design (S)**:

- **Review Questions**:

1. What study design is outlined?

2. Does the study design match the **S** criterion as per the PICOS requirements?

- **Instructions**:

- Mark **TRUE** if the study design fits inclusion (RCT).

- Mark **FALSE** if it does not meet the criteria.

- **Provide a brief explanation** for why the particular design meets or does not meet the criteria.

\# Final Decision

- If **all PICOS criteria** are marked as **TRUE**, the **FINAL DECISION** is **TRUE**.

- If **any of the PICOS criteria** is marked as **FALSE**, the **FINAL DECISION** must be **FALSE**.

- Always provide a reason for the **FINAL DECISION**.

**Follow the output template precisely while responding.**

"""

\# User Prompt

user_prompt = """

\### PICOS Criteria

**Population (P)**:

Adults aged ≥18 years with established atherosclerotic cardiovascular disease (ASCVD) in secondary prevention.

Includes patients with any prior conditions of:

1) Coronary artery disease (e.g., myocardial infarction, stable angina)

2) Ischemic stroke

3) Peripheral arterial disease

**Intervention & Comparator (I&C)**:

The study must compare two different **statin treatment categories**.

Eligible categories are:

1. Moderate-intensity statins (MIS) monotherapy

2. MIS + ezetimibe

3. Treat-to-target statins

4. High-intensity statins (HIS)

Valid comparisons:

- Any comparison between **two different categories** above.

- If multiple pairs are reported, the study is eligible if **at least one** valid pair appears.

Important rule:

- The comparison must be clearly about **statin regimens or statin intensity**.

- Do NOT count comparisons based on diagnosis, disease severity, procedures, timing, or risk groups

(e.g., NSTE-ACS vs STEMI, early vs late PCI).

Exclusions:

- HIS + ezetimibe

- HIS vs HIS (same category)

- Statin users vs non-users

- Studies that do not specify statin regimen categories

**Outcome (O)**:

Include any of the following:

- Major Adverse Cardiovascular Events (MACE), defined as composite of these conditions

(i) all-cause death, ii) myocardial infarction, iii) ischemic stroke, iv) any coronary revascularization)

- any of individual outcomes, including, cardiovascular death, LDL-C reduction, heart failure, statin-induced muscle symptoms (SIMS), rhabdomyolysis, new-onset diabetes, drug-induced liver injury, hemorrhagic stroke

**Study Design (S)**:

Include:

- Observational studies (prospective or retrospective cohorts, registry studies)

- Studies described as “target trial,” “target trial emulation,”

“trial emulation,” “emulated trial,” “emulated RCT,” or “emulating a target trial”

Exclude:

- Case-control studies

- Randomized controlled trials (RCTs)

- Clinical trials

\### Input

**Title and Abstract**:

```

{INPUT}

```

\# Notes

- Always lean towards **inclusion** unless the information explicitly indicates exclusion.

- If unsure, assume it might be clarified in the full text and mark as **TRUE**.

""".format(INPUT = INPUT)

prompt_dict = {'system_prompt': system_prompt, 'user_prompt': user_prompt}

return prompt_dict
