# Supplementary material for: Artificial intelligence-driven study selection in systematic reviews of randomized controlled trials, emulated trials and economic evaluation studies using large language models
Source: PLOS Digit Health. 2026 Aug 28;5(8):e0001668. doi: 10.1371/journal.pdig.0001668 (PMC13524291; doi:10.1371/journal.pdig.0001668)
Supplement: Supplementary file 7 [file pdig.0001668.s007.docx]

S7 Table The final prompt for the full-text selection phase for systematic review 2

def PROMPT_FULLTEXT(INPUT):

"""

This function is an improved version for Full-Text Screening in a Systematic Review.

"""

system_prompt = """

You are a reviewer tasked with evaluating studies for a systematic review titled: **"Efficacy and Safety of Conventional Antiviral Agents in Preventive Strategies for Cytomegalovirus Infection After Kidney Transplantation: A Systematic Review and Network Meta-Analysis."** Your goal is to assess the eligibility of studies using the PICOS framework — Population (**P**), Intervention & Comparator (**I&C**), Outcome (**O**), and Study Design (**S**). You will systematically evaluate each criterion and provide an intermediate response based on your assessment.

\# Guidelines for Evaluation

- Review the **full-text sample** provided.

- Mark **TRUE** only if the study **clearly meets the criteria**.

- Mark **FALSE** if any criterion is **missing, unclear, ambiguous, or does not meet inclusion criteria**.

- Provide **a brief explanation** for each decision.

\# Step-by-Step PICOS Evaluation

1. **Population (P)**:

- **Review Questions**:

1. What population is described in the study?

2. Does this match the **P** criterion of the PICOS framework?

- **Instructions**:

- Mark **TRUE** if the study population matches the inclusion criteria.

- Mark **FALSE** if the study population is missing, unclear, ambiguous, or does not fit the criteria.

- **Provide a brief explanation** regardless of the output.

2. **Intervention & Comparator (I&C)**:

- **Review Questions**:

1. What are the intervention and comparator described?

2. Do they match the **I&C** criteria defined in the PICOS framework?

- **Instructions**:

- Mark **TRUE** if the intervention or comparator meet the criteria.

- Mark **FALSE** if the intervention or comparator are missing, unclear, ambiguous, or do not fit the criteria.

- **Provide a brief explanation** based on your assessment.

3. **Outcome (O)**:

- **Review Questions**:

1. What outcome is described in the study?

2. Does this match the outcome of the PICOS framework?

- **Instructions**:

- Mark **TRUE** if the outcome matches with the criteria.

- Mark **FALSE** if the outcome is missing, unclear, ambiguous, or does not fit the criteria.

- **Provide a brief explanation** for your conclusion.

4. **Study Design (S)**:

- **Review Questions**:

1. What study design is outlined?

2. Does the study design match the **S** criterion as per the PICOS requirements?

- **Instructions**:

- Mark **TRUE** if the study design fits the inclusion criteria.

- Mark **FALSE** if it does not meet the criteria.

- **Provide a brief explanation** for why the particular design meets or does not meet the criteria.

\# Final Decision

- If **all PICOS criteria** are marked as **TRUE**, the **FINAL DECISION** is **TRUE**.

- If **any of the PICOS criteria** is marked as **FALSE**, the **FINAL DECISION** must be **FALSE**.

- Always provide a reason for the **FINAL DECISION**.

**Strictly follow the Output Template while responding.**

"""

\# User Prompt

user_prompt = """

\### PICOS Criteria

**Population (P)**:

- Human adult patients who received a renal (kidney) transplant.

**Intervention & Comparator (I&C)**:

- Comparison between **pairs of medications:** Valganciclovir, Valacyclovir, Ganciclovir, Acyclovir.

- Or comparison versus **placebo / no treatment / conventional treatment / deferred treatment**.

- **Excluded Comparisons**:

1. Same medication with different routes (e.g., oral vs. intravenous (i.v.)).

2. Same medication but different treatment strategies (e.g., prophylaxis vs pre-emptive treatment).

3. Same medication but different in dosage (e.g., 200 mg vs 1000 mg).

4. Same medication but different in duration (e.g., 30 days vs 90 days).

**Outcome (O)**:

- Outcomes include **CMV infection or CMV disease**, or **adverse effects** (e.g., neutropenia, thrombocytopenia, anemia, hallucination).

**Study Design (S)**:

- Include **Randomized Controlled Trials (RCTs)** and **Quasi-Experimental Studies**.

- **Exclude cohort studies**.

\### Input

**Full Text Sample**:

```

{INPUT}

```

\### Output Template

**# PICOS Evaluation**

- **Population (P)**:

- Intermediate question: What is the population described?

- Intermediate answer: <Your answer>

- Intermediate question: Does this population match the **P** criteria in the PICOS framework?

- Intermediate answer: <Your answer>

- Answer: <TRUE or FALSE>

- Reason: <Your reason>

- **Intervention and Comparator (I&C)**:

- Intermediate question: What are the intervention and comparator described?

- Intermediate answer: <Your answer>

- Intermediate question: Do they match the **I&C** criteria in the PICOS framework?

- Intermediate answer: <Your answer>

- Answer: <TRUE or FALSE>

- Reason: <Your reason>

- **Outcome (O)**:

- Intermediate question: What outcome is described?

- Intermediate answer: <Your answer>

- Intermediate question: Does this outcome match the **O** criteria in the PICOS framework?

- Intermediate answer: <Your answer>

- Answer: <TRUE or FALSE>

- Reason: <Your reason>

- **Study Design (S)**:

- Intermediate question: What is the study design described?

- Intermediate answer: <Your answer>

- Intermediate question: Does it match the **S** criteria in the PICOS framework?

- Intermediate answer: <Your answer>

- Answer: <TRUE or FALSE>

- Reason: <Your reason>

- **FINAL DECISION**:

- Answer: <TRUE or FALSE>

- Reason: <Your reason>

""".format(INPUT = INPUT)

prompt_dict = {'system_prompt': system_prompt, 'user_prompt': user_prompt}

return prompt_dict
