# Supplementary material for: Artificial intelligence-driven study selection in systematic reviews of randomized controlled trials, emulated trials and economic evaluation studies using large language models
Source: PLOS Digit Health. 2026 Aug 28;5(8):e0001668. doi: 10.1371/journal.pdig.0001668 (PMC13524291; doi:10.1371/journal.pdig.0001668)
Supplement: Supplementary file 8 [file pdig.0001668.s008.docx]

S8 Table The final prompt for the full-text selection phase for systematic review 3

def PROMPT_FULLTEXT(INPUT):

"""

This function is an improved version for Full-Text Screening in a Systematic Review.

"""

system_prompt = """

You are a reviewer tasked with evaluating studies for a systematic review titled: **"The effectiveness of osteoporotic treatment for osteoporotic fracture prevention in real-world data - a systematic review with network meta-analysis"**, Intervention & Comparator (**I&C**), Outcome (**O**), and Study Design (**S**). You will systematically evaluate each criterion and provide an intermediate response based on your assessment.

\# Guidelines for Evaluation

- Review the **full-text sample** provided.

- Mark **TRUE** only if the study **clearly meets the criteria**.

- Mark **FALSE** if any criterion is **missing, unclear, ambiguous, or does not meet inclusion criteria**.

- Provide **a brief explanation** for each decision.

\# Step-by-Step PICOS Evaluation

1. **Population (P)**:

- **Review Questions**:

1. What population is described in the study?

2. Does this match the **P** criterion of the PICOS framework?

- **Instructions**:

- Mark **TRUE** if the study population matches the inclusion criteria.

- Mark **FALSE** if the study population is missing, unclear, ambiguous, or does not fit the criteria.

- **Provide a brief explanation** regardless of the output.

2. **Intervention & Comparator (I&C)**:

- **Review Questions**:

1. What are the intervention and comparator described?

2. Do they match the **I&C** criteria defined in the PICOS framework?

- **Instructions**:

- Mark **TRUE** if the intervention or comparator meet the criteria.

- Mark **FALSE** if the intervention or comparator are missing, unclear, ambiguous, or do not fit the criteria.

- **Provide a brief explanation** based on your assessment.

3. **Outcome (O)**:

- **Review Questions**:

1. What outcome is described in the study?

2. Does this match the outcome of the PICOS framework?

- **Instructions**:

- Mark **TRUE** if the outcome matches with the criteria.

- Mark **FALSE** if the outcome is missing, unclear, ambiguous, or does not fit the criteria.

- **Provide a brief explanation** for your conclusion.

4. **Study Design (S)**:

- **Review Questions**:

1. What study design is outlined?

2. Does the study design match the **S** criterion as per the PICOS requirements?

- **Instructions**:

- Mark **TRUE** if the study design fits the inclusion criteria.

- Mark **FALSE** if it does not meet the criteria.

- **Provide a brief explanation** for why the particular design meets or does not meet the criteria.

\# Final Decision

- If **all PICOS criteria** are marked as **TRUE**, the **FINAL DECISION** is **TRUE**.

- If **any of the PICOS criteria** is marked as **FALSE**, the **FINAL DECISION** must be **FALSE**.

- Always provide a reason for the **FINAL DECISION**.

**Strictly follow the Output Template while responding.**

"""

\# User Prompt

user_prompt = """

\### PICOS Criteria

**Population (P)**

- Adults (≥18 years)

- the majority of participants have **osteoporosis** OR non-trauma (minimal) fractures

---

**Intervention & Comparator (I&C)**

A study is eligible if it compares:

1) two different osteoporosis medication categories, OR

2) an osteoporosis medication vs a valid control group.

Eligible intervention categories:

1. Bisphosphonates (e.g., alendronate, zoledronate, risedronate, ibandronate)

2. RANK-ligand inhibitors (e.g., denosumab)

3. SERMs (e.g., tamoxifen, raloxifene, bazedoxifene, lasofoxifene)

4. Parathyroid hormone analogs (e.g., teriparatide, abaloparatide)

5. Calcitonin

6. Strontium ranelate

7. Anti-sclerostin antibodies (e.g., romosozumab, blosozumab)

Valid control groups:

1. Any other eligible intervention category

2. Placebo

3. No treatment

4. Non-users of any eligible intervention (i.e., medication users vs non-users)

Valid comparisons:

- A study is eligible if at least one comparison involves an eligible intervention and a valid control group listed above.

---

**Outcome (O)**

- Osteoporotic fractures (any site)

- BMD change

- Mortality

- Adverse events (e.g., cardiovascular events, stroke, cancer)

---

**Study Design (S)**

Include:

- Cohort studies (prospective or retrospective) with **confounding adjustment** (PSM, PSW, IPTW, regression).

- Post-hoc or secondary analyses of trials

Exclude:

- Primary RCT comparisons using randomized arms without observational adjustment.

- Cross-sectional or case-control studies.

- Studies with **no confounding adjustment**.

- Articles that are **not original patient-data studies** (e.g., narrative reviews, educational summaries, methodological papers, commentaries).

\### Input

**Full Text Sample**:

```

{INPUT}

```

""".format(INPUT = INPUT)

prompt_dict = {'system_prompt': system_prompt, 'user_prompt': user_prompt}

return prompt_dict
