# Supplementary material for: Artificial intelligence-driven study selection in systematic reviews of randomized controlled trials, emulated trials and economic evaluation studies using large language models
Source: PLOS Digit Health. 2026 Aug 28;5(8):e0001668. doi: 10.1371/journal.pdig.0001668 (PMC13524291; doi:10.1371/journal.pdig.0001668)
Supplement: Supplementary file 11 [file pdig.0001668.s011.docx]

S11 Table Performance and pairwise comparison of large language models in the title and abstract screening phase

| **SR** | **LLM** | **%Recall**  **(95% CI)** | **%Recall**  **p-value** | **%RW**  **(95% CI)** | **%RW**  **p-value** | **%Precision**  **(95% CI)** | **%FNR**  **(95% CI)** | **NNS_1_** | **NNR** |
| --- | --- | --- | --- | --- | --- | --- | --- | --- | --- |
| SR1 (2020) | Gemini 2.0 | 100.00 (77.29-100.00) | 1.000(vs.Llama) 0.883(vs.Qwen) | 97.03 (96.50-97.56) | 0.854(vs.Llama) <0.001*(vs.Qwen) | 15.38 (8.84-21.92) | 0.00 (0.00-22.71) | 117 | 6.50 |
|  | Llama 3.1 | 100.00 (77.29-100.00) | 1.000(vs.Gemini) 0.883(vs.Qwen) | 97.10 (96.58-97.62) | 0.854(vs.Gemini) <0.001*(vs.Qwen) | 15.79 (9.10-22.48) | 0.00 (0.00-22.71) | 114 | 6.33 |
|  | Qwen 2.5 | 88.89 (74.37-100.00) | 0.883(vs.Gemini) 0.883(vs.Llama) | 99.19 (98.91-99.47) | <0.001*(vs.Gemini) <0.001*(vs.Llama) | 50.00 (32.68-67.32) | 11.11 (0.00-25.63) | 32 | 2.00 |
| SR2 (2021) | Gemini 2.0 | 90.48 (77.93-100.00) | 0.547(vs.Llama) 0.373(vs.Qwen) | 89.72 (88.64-90.80) | 0.203(vs.Llama) <0.001*(vs.Qwen) | 6.05 (3.41-8.69) | 9.52 (0.00-22.07) | 314 | 16.53 |
|  | Llama 3.1 | 95.24 (86.13-100.00) | 0.547(vs.Gemini) 0.143(vs.Qwen) | 88.71 (87.59-89.83) | 0.203(vs.Gemini) <0.001*(vs.Qwen) | 5.80 (3.33-8.27) | 4.76 (0.00-13.87) | 345 | 17.24 |
|  | Qwen 2.5 | 80.95 (64.15-97.75) | 0.373(vs.Gemini) 0.143(vs.Llama) | 98.17 (97.69-98.65) | <0.001*(vs.Gemini) <0.001*(vs.Llama) | 30.36 (18.32-42.40) | 19.05 (2.25-35.85) | 56 | 3.29 |
| SR3 (2024) | Gemini 2.0 | 100.00 (89.31-100.00) | 1.000(vs.Llama) 0.110(vs.Qwen) | 77.10 (73.66-80.54) | 0.049*(vs.Llama) 0.033*(vs.Qwen) | 33.59 (25.50-41.68) | 0.00 (0.00-10.69) | 131 | 2.98 |
|  | Llama 3.1 | 100.00 (89.31-100.00) | 1.000(vs.Gemini) 0.110(vs.Qwen) | 72.03 (68.35-75.71) | 0.049*(vs.Gemini) <0.001*(vs.Qwen) | 27.50 (20.58-34.42) | 0.00 (0.00-10.69) | 160 | 3.64 |
|  | Qwen 2.5 | 81.82 (70.42-93.22) | 0.110(vs.Gemini) 0.110(vs.Llama) | 82.17 (79.03-85.31) | 0.033*(vs.Gemini) <0.001*(vs.Llama) | 35.29 (26.02-44.56) | 18.18 (6.78-29.58) | 102 | 2.83 |
| SR4 (2025) | Gemini 2.0 | 100.00 (85.05-100.00) | 0.688(vs.Llama) 0.144(vs.Qwen) | 95.33 (94.56-96.10) | <0.001*(vs.Llama) 0.020*(vs.Qwen) | 22.22 (15.21-29.23) | 0.00 (0.00-14.95) | 135 | 4.50 |
|  | Llama 3.1 | 90.00 (79.26-100.00) | 0.688(vs.Gemini) 0.159(vs.Qwen) | 90.73 (89.67-91.79) | <0.001*(vs.Gemini) <0.001*(vs.Qwen) | 10.07 (6.47-13.67) | 10.00 (0.00-20.74) | 268 | 9.93 |
|  | Qwen 2.5 | 76.67 (61.54-91.80) | 0.144(vs.Gemini) 0.159(vs.Llama) | 96.54 (95.87-97.21) | 0.020*(vs.Gemini) <0.001*(vs.Llama) | 23.00 (14.75-31.25) | 23.33 (8.20-38.46) | 100 | 4.35 |
| SR5 (2026) | Gemini 2.0 | 100.00 (92.82-100.00) | 0.799(vs.Llama) 0.020*(vs.Qwen) | 65.21 (60.32-70.10) | 0.355(vs.Llama) 0.001*(vs.Qwen) | 53.54 (44.87-62.21) | 0.00 (0.00-7.18) | 127 | 1.87 |
|  | Llama 3.1 | 98.53 (95.67-100.00) | 0.799(vs.Gemini) <0.001*(vs.Qwen) | 61.92 (56.94-66.90) | 0.355(vs.Gemini) <0.001*(vs.Qwen) | 48.20 (39.89-56.51) | 1.47 (0.00-4.33) | 139 | 2.07 |
|  | Qwen 2.5 | 82.35 (73.29-91.41) | 0.020*(vs.Gemini) <0.001*(vs.Llama) | 76.16 (71.79-80.53) | 0.001*(vs.Gemini) <0.001*(vs.Llama) | 64.37 (54.31-74.43) | 17.65 (8.59-26.71) | 87 | 1.55 |

* Statistically significant difference

Note: Wald 95% CIs are used by default. Wilson 95% CIs are used only for perfect evaluation scores, so boundary estimates retain finite-sample uncertainty.
