# Supplementary material for: Artificial intelligence-driven study selection in systematic reviews of randomized controlled trials, emulated trials and economic evaluation studies using large language models
Source: PLOS Digit Health. 2026 Aug 28;5(8):e0001668. doi: 10.1371/journal.pdig.0001668 (PMC13524291; doi:10.1371/journal.pdig.0001668)
Supplement: Supplementary file 12 [file pdig.0001668.s012.docx]

S12 Table The confusion and performance metrics of large language models in the full text selection phase

| **SR** | **LLM** | **Round** | **Test data** | **TP** | **FP** | **FN** | **TN** | **Precision** | **Recall** | **FNR** | **F1** | **%RW** | **NNS_2_** | **NNR** | **Time/Doc** |
| --- | --- | --- | --- | --- | --- | --- | --- | --- | --- | --- | --- | --- | --- | --- | --- |
| SR1 | Gemini 2.0 Flash | 1 | 117 | 18 | 26 | 0 | 73 | 40.91 (26.38-55.44) | 100.00 (77.29-100.00) | 0.00 (0.00-22.71) | 58.06 (45.78-70.34) | 98.88 (98.55-99.21) | 44 | 2.44 | 6s |
|  |  | 2 | 117 | 18 | 25 | 0 | 74 | 41.86 (27.11-56.61) | 100.00 (77.29-100.00) | 0.00 (0.00-22.71) | 59.02 (46.68-71.36) | 98.91 (98.59-99.23) | 43 | 2.39 | 6s |
|  |  | 3 | 117 | 18 | 26 | 0 | 73 | 40.91 (26.38-55.44) | 100.00 (77.29-100.00) | 0.00 (0.00-22.71) | 58.06 (45.78-70.34) | 98.88 (98.55-99.21) | 44 | 2.44 | 6s |
|  | Llama 3.1 | 1 | 114 | 18 | 84 | 0 | 12 | 17.65 (10.25-25.05) | 100.00 (77.29-100.00) | 0.00 (0.00-22.71) | 30.00 (21.80-38.20) | 97.41 (96.91-97.91) | 102 | 5.67 | 2m 29s |
|  |  | 2 | 114 | 18 | 84 | 0 | 12 | 17.65 (10.25-25.05) | 100.00 (77.29-100.00) | 0.00 (0.00-22.71) | 30.00 (21.80-38.20) | 97.41 (96.91-97.91) | 102 | 5.67 | 2m 14s |
|  |  | 3 | 114 | 18 | 84 | 0 | 12 | 17.65 (10.25-25.05) | 100.00 (77.29-100.00) | 0.00 (0.00-22.71) | 30.00 (21.80-38.20) | 97.41 (96.91-97.91) | 102 | 5.67 | 2m 14s |
|  | Qwen 2.5 | 1 | 34 | 14 | 8 | 4 | 8 | 63.64 (43.54-83.74) | 77.78 (58.57-96.99) | 22.22 (3.01-41.43) | 70.00 (55.80-84.20) | 99.44 (99.21-99.67) | 22 | 1.57 | 3m 32s |
|  |  | 2 | 34 | 14 | 8 | 4 | 8 | 63.64 (43.54-83.74) | 77.78 (58.57-96.99) | 22.22 (3.01-41.43) | 70.00 (55.80-84.20) | 99.44 (99.21-99.67) | 22 | 1.57 | 3m 32s |
|  |  | 3 | 34 | 14 | 8 | 4 | 8 | 63.64 (43.54-83.74) | 77.78 (58.57-96.99) | 22.22 (3.01-41.43) | 70.00 (55.80-84.20) | 99.44 (99.21-99.67) | 22 | 1.57 | 3m 32s |
| **SR** | **LLM** | **Round** | **Test data** | **TP** | **FP** | **FN** | **TN** | **Precision** | **Recall** | **FNR** | **F1** | **%RW** | **NNS_2_** | **NNR** | **Time/Doc** |
| SR2 | Gemini 2.0 Flash | 1 | 316 | 19 | 46 | 2 | 249 | 29.23 (18.17-40.29) | 90.48 (77.93-100.00) | 9.52 (0.00-22.07) | 44.19 (33.69-54.69) | 97.87 (97.36-98.38) | 65 | 3.42 | 7s |
|  |  | 2 | 316 | 19 | 45 | 2 | 250 | 29.69 (18.50-40.88) | 90.48 (77.93-100.00) | 9.52 (0.00-22.07) | 44.71 (34.14-55.28) | 97.91 (97.40-98.42) | 64 | 3.37 | 8s |
|  |  | 3 | 316 | 19 | 46 | 2 | 249 | 29.23 (18.17-40.29) | 90.48 (77.93-100.00) | 9.52 (0.00-22.07) | 44.19 (33.69-54.69) | 97.87 (97.36-98.38) | 65 | 3.42 | 8s |
|  | Llama 3.1 | 1 | 346 | 21 | 229 | 0 | 96 | 8.40 (4.96-11.84) | 100.00 (79.90-100.00) | 0.00 (0.00-20.10) | 15.50 (11.19-19.81) | 91.82 (90.85-92.79) | 250 | 11.90 | 2m 54s |
|  |  | 2 | 346 | 21 | 229 | 0 | 96 | 8.40 (4.96-11.84) | 100.00 (79.90-100.00) | 0.00 (0.00-20.10) | 15.50 (11.19-19.81) | 91.82 (90.85-92.79) | 250 | 11.90 | 2m 54s |
|  |  | 3 | 346 | 21 | 229 | 0 | 96 | 8.40 (4.96-11.84) | 100.00 (79.90-100.00) | 0.00 (0.00-20.10) | 15.50 (11.19-19.81) | 91.82 (90.85-92.79) | 250 | 11.90 | 2m 54s |
|  | Qwen 2.5 | 1 | 60 | 17 | 26 | 4 | 13 | 39.53 (24.92-54.14) | 80.95 (64.15-97.75) | 19.05 (2.25-35.85) | 53.12 (40.89-65.35) | 98.59 (98.17-99.01) | 43 | 2.53 | 2m 58s |
|  |  | 2 | 60 | 17 | 26 | 4 | 13 | 39.53 (24.92-54.14) | 80.95 (64.15-97.75) | 19.05 (2.25-35.85) | 53.12 (40.89-65.35) | 98.59 (98.17-99.01) | 43 | 2.53 | 2m 58s |
|  |  | 3 | 60 | 17 | 26 | 4 | 13 | 39.53 (24.92-54.14) | 80.95 (64.15-97.75) | 19.05 (2.25-35.85) | 53.12 (40.89-65.35) | 98.59 (98.17-99.01) | 43 | 2.53 | 2m 59s |
| **SR** | **LLM** | **Round** | **Test data** | **TP** | **FP** | **FN** | **TN** | **Precision** | **Recall** | **FNR** | **F1** | **%RW** | **NNS_2_** | **NNR** | **Time/Doc** |
| SR3 | Gemini 2.0 Flash | 1 | 131 | 41 | 41 | 3 | 46 | 50.00 (39.18-60.82) | 93.18 (85.73-100.00) | 6.82 (0.00-14.27) | 65.08 (56.76-73.40) | 85.66 (82.79-88.53) | 82 | 2.00 | 5s |
|  |  | 2 | 131 | 39 | 40 | 5 | 47 | 49.37 (38.35-60.39) | 88.64 (79.26-98.02) | 11.36 (1.98-20.74) | 63.41 (54.90-71.92) | 86.19 (83.36-89.02) | 79 | 2.03 | 5s |
|  |  | 3 | 131 | 40 | 38 | 4 | 49 | 51.28 (40.19-62.37) | 90.91 (82.42-99.40) | 9.09 (0.60-17.58) | 65.57 (57.14-74.00) | 86.36 (83.55-89.17) | 78 | 1.95 | 6s |
|  | Llama 3.1 | 1 | 160 | 43 | 75 | 1 | 41 | 36.44 (27.76-45.12) | 97.73 (93.33-100.00) | 2.27 (0.00-6.67) | 53.09 (45.41-60.77) | 79.37 (76.05-82.69) | 118 | 2.74 | 3m 31s |
|  |  | 2 | 160 | 43 | 75 | 1 | 41 | 36.44 (27.76-45.12) | 97.73 (93.33-100.00) | 2.27 (0.00-6.67) | 53.09 (45.41-60.77) | 79.37 (76.05-82.69) | 118 | 2.74 | 3m 31s |
|  |  | 3 | 160 | 43 | 75 | 1 | 41 | 36.44 (27.76-45.12) | 97.73 (93.33-100.00) | 2.27 (0.00-6.67) | 53.09 (45.41-60.77) | 79.37 (76.05-82.69) | 118 | 2.74 | 3m 31s |
|  | Qwen 2.5 | 1 | 110 | 38 | 36 | 6 | 30 | 51.35 (39.96-62.74) | 86.36 (76.22-96.50) | 13.64 (3.50-23.78) | 64.41 (55.77-73.05) | 87.06 (84.31-89.81) | 74 | 1.95 | 3m 34s |
|  |  | 2 | 110 | 38 | 36 | 6 | 30 | 51.35 (39.96-62.74) | 86.36 (76.22-96.50) | 13.64 (3.50-23.78) | 64.41 (55.77-73.05) | 87.06 (84.31-89.81) | 74 | 1.95 | 3m 34s |
|  |  | 3 | 110 | 38 | 36 | 6 | 30 | 51.35 (39.96-62.74) | 86.36 (76.22-96.50) | 13.64 (3.50-23.78) | 64.41 (55.77-73.05) | 87.06 (84.31-89.81) | 74 | 1.95 | 3m 34s |
| **SR** | **LLM** | **Round** | **Test data** | **TP** | **FP** | **FN** | **TN** | **Precision** | **Recall** | **FNR** | **F1** | **%RW** | **NNS_2_** | **NNR** | **Time/Doc** |
| SR4 | Gemini 2.0 Flash | 1 | 135 | 26 | 22 | 4 | 83 | 54.17 (40.07-68.27) | 86.67 (74.51-98.83) | 13.33 (1.17-25.49) | 66.67 (56.21-77.13) | 98.34 (97.87-98.81) | 48 | 1.85 | 5s |
|  |  | 2 | 135 | 25 | 22 | 5 | 83 | 53.19 (38.92-67.46) | 83.33 (69.99-96.67) | 16.67 (3.33-30.01) | 64.94 (54.28-75.60) | 98.37 (97.91-98.83) | 47 | 1.88 | 5s |
|  |  | 3 | 135 | 27 | 23 | 3 | 82 | 54.00 (40.19-67.81) | 90.00 (79.26-100.00) | 10.00 (0.00-20.74) | 67.50 (57.24-77.76) | 98.27 (97.79-98.75) | 50 | 1.85 | 5s |
|  | Llama 3.1 | 1 | 271 | 28 | 140 | 2 | 101 | 16.67 (11.03-22.31) | 93.33 (84.40-100.00) | 6.67 (0.00-15.60) | 28.28 (22.01-34.55) | 94.19 (93.34-95.04) | 168 | 6.00 | 3m 1s |
|  |  | 2 | 271 | 28 | 140 | 2 | 101 | 16.67 (11.03-22.31) | 93.33 (84.40-100.00) | 6.67 (0.00-15.60) | 28.28 (22.01-34.55) | 94.19 (93.34-95.04) | 168 | 6.00 | 3m 1s |
|  |  | 3 | 271 | 28 | 140 | 2 | 101 | 16.67 (11.03-22.31) | 93.33 (84.40-100.00) | 6.67 (0.00-15.60) | 28.28 (22.01-34.55) | 94.19 (93.34-95.04) | 168 | 6.00 | 3m 1s |
|  | Qwen 2.5 | 1 | 107 | 20 | 6 | 10 | 71 | 76.92 (60.72-93.12) | 66.67 (49.80-83.54) | 33.33 (16.46-50.20) | 71.43 (59.60-83.26) | 99.10 (98.76-99.44) | 26 | 1.30 | 4m 11s |
|  |  | 2 | 107 | 20 | 6 | 10 | 71 | 76.92 (60.72-93.12) | 66.67 (49.80-83.54) | 33.33 (16.46-50.20) | 71.43 (59.60-83.26) | 99.10 (98.76-99.44) | 26 | 1.30 | 4m 11s |
|  |  | 3 | 107 | 20 | 6 | 10 | 71 | 76.92 (60.72-93.12) | 66.67 (49.80-83.54) | 33.33 (16.46-50.20) | 71.43 (59.60-83.26) | 99.10 (98.76-99.44) | 26 | 1.30 | 4m 11s |
| **SR** | **LLM** | **Round** | **Test data** | **TP** | **FP** | **FN** | **TN** | **Precision** | **Recall** | **FNR** | **F1** | **%RW** | **NNS_2_** | **NNR** | **Time/Doc** |
| SR5 | Gemini 2.0 Flash | 1 | 127 | 68 | 14 | 0 | 45 | 82.93 (74.79-91.07) | 100.00 (92.82-100.00) | 0.00 (0.00-7.18) | 90.67 (86.02-95.32) | 77.53 (73.25-81.81) | 82 | 1.21 | 5s |
|  |  | 2 | 127 | 68 | 17 | 0 | 42 | 80.00 (71.50-88.50) | 100.00 (92.82-100.00) | 0.00 (0.00-7.18) | 88.89 (83.91-93.87) | 76.71 (72.37-81.05) | 85 | 1.25 | 5s |
|  |  | 3 | 127 | 68 | 15 | 0 | 44 | 81.93 (73.65-90.21) | 100.00 (92.82-100.00) | 0.00 (0.00-7.18) | 90.07 (85.30-94.84) | 77.26 (72.96-81.56) | 83 | 1.22 | 4s |
|  | Llama 3.1 | 1 | 140 | 64 | 34 | 4 | 38 | 65.31 (55.89-74.73) | 94.12 (88.53-99.71) | 5.88 (0.29-11.47) | 77.11 (70.72-83.50) | 73.15 (68.60-77.70) | 98 | 1.53 | 2m 43s |
|  |  | 2 | 140 | 64 | 34 | 4 | 38 | 65.31 (55.89-74.73) | 94.12 (88.53-99.71) | 5.88 (0.29-11.47) | 77.11 (70.72-83.50) | 73.15 (68.60-77.70) | 98 | 1.53 | 2m 43s |
|  |  | 3 | 140 | 64 | 34 | 4 | 38 | 65.31 (55.89-74.73) | 94.12 (88.53-99.71) | 5.88 (0.29-11.47) | 77.11 (70.72-83.50) | 73.15 (68.60-77.70) | 98 | 1.53 | 2m 43s |
|  | Qwen 2.5 | 1 | 99 | 61 | 9 | 7 | 22 | 87.14 (79.30-94.98) | 89.71 (82.49-96.93) | 10.29 (3.07-17.51) | 88.41 (83.07-93.75) | 80.82 (76.78-84.86) | 70 | 1.15 | 3m 59s |
|  |  | 2 | 99 | 61 | 9 | 7 | 22 | 87.14 (79.30-94.98) | 89.71 (82.49-96.93) | 10.29 (3.07-17.51) | 88.41 (83.07-93.75) | 80.82 (76.78-84.86) | 70 | 1.15 | 3m 59s |
|  |  | 3 | 99 | 61 | 9 | 7 | 22 | 87.14 (79.30-94.98) | 89.71 (82.49-96.93) | 10.29 (3.07-17.51) | 88.41 (83.07-93.75) | 80.82 (76.78-84.86) | 70 | 1.15 | 3m 59s |

doc, document; FN, false negatives; FP, false positives; LLM, large language model; m, minutes NNR, number needed to read; NNS_2_, number needed to screen in full text selection phase; SR, systematic review; Time/doc, average time per document; TN, true negatives; TP, true positives; %RW, % reduced workload;

Note*: Wald 95% CIs are used by default. Wilson 95% CIs are used only for perfect evaluation scores, so boundary estimates retain finite-sample uncertainty.

Note**: Full-text evaluation metrics were calculated using model-specific full-text datasets generated from the outputs of title/abstract screening phase (i.e., TP, FP, and FN). Therefore, the number and composition of studies evaluated at the full-text phase differed across LLMs and review datasets.
