# Supplementary material for: Artificial intelligence-driven study selection in systematic reviews of randomized controlled trials, emulated trials and economic evaluation studies using large language models
Source: PLOS Digit Health. 2026 Aug 28;5(8):e0001668. doi: 10.1371/journal.pdig.0001668 (PMC13524291; doi:10.1371/journal.pdig.0001668)
Supplement: Supplementary file 13 [file pdig.0001668.s013.docx]

S13 Table Performance and pairwise comparison of large language models in the full-text selection phase

| **SR** | **LLM** | **%Recall**  **(95% CI)** | **%Recall**  **p-value** | **%RW**  **(95% CI)** | **%RW**  **p-value** | **%Precision**  **(95% CI)** | **%FNR**  **(95% CI)** | **NNS_2_** | **NNR** |
| --- | --- | --- | --- | --- | --- | --- | --- | --- | --- |
| SR1 (2020) | Gemini 2.0 | 100.00 (77.29-100.00) | 1.000(vs.Llama) 0.430(vs.Qwen) | 98.91 (98.59-99.23) | <0.001*(vs.Llama) 0.009*(vs.Qwen) | 41.86 (27.11-56.61) | 0.00 (0.00-22.71) | 43 | 2.39 |
|  | Llama 3.1 | 100.00 (77.29-100.00) | 1.000(vs.Gemini) 0.430(vs.Qwen) | 97.41 (96.91-97.91) | <0.001*(vs.Gemini) <0.001*(vs.Qwen) | 17.65 (10.25-25.05) | 0.00 (0.00-22.71) | 102 | 5.67 |
|  | Qwen 2.5 | 77.78 (58.57-96.99) | 0.430(vs.Gemini) 0.430(vs.Llama) | 99.44 (99.21-99.67) | 0.009*(vs.Gemini) <0.001*(vs.Llama) | 63.64 (43.54-83.74) | 22.22 (3.01-41.43) | 22 | 1.57 |
| SR2 (2021) | Gemini 2.0 | 90.48 (77.93-100.00) | 0.898(vs.Llama) 0.373(vs.Qwen) | 97.91 (97.40-98.42) | <0.001*(vs.Llama) 0.043*(vs.Qwen) | 29.69 (18.50-40.88) | 9.52 (0.00-22.07) | 64 | 3.37 |
|  | Llama 3.1 | 100.00 (79.90-100.00) | 0.898(vs.Gemini) 0.452(vs.Qwen) | 91.82 (90.85-92.79) | <0.001*(vs.Gemini) <0.001*(vs.Qwen) | 8.40 (4.96-11.84) | 0.00 (0.00-20.10) | 250 | 11.90 |
|  | Qwen 2.5 | 80.95 (64.15-97.75) | 0.373(vs.Gemini) 0.452(vs.Llama) | 98.59 (98.17-99.01) | 0.043*(vs.Gemini) <0.001*(vs.Llama) | 39.53 (24.92-54.14) | 19.05 (2.25-35.85) | 43 | 2.53 |
| SR3 (2024) | Gemini 2.0 | 93.18 (85.73-100.00) | 0.303(vs.Llama) 0.288(vs.Qwen) | 85.66 (82.79-88.53) | 0.005*(vs.Llama) 0.490(vs.Qwen) | 50.00 (39.18-60.82) | 6.82 (0.00-14.27) | 82 | 2.00 |
|  | Llama 3.1 | 97.73 (93.33-100.00) | 0.303(vs.Gemini) 0.044*(vs.Qwen) | 79.37 (76.05-82.69) | 0.005*(vs.Gemini) <0.001*(vs.Qwen) | 36.44 (27.76-45.12) | 2.27 (0.00-6.67) | 118 | 2.74 |
|  | Qwen 2.5 | 86.36 (76.22-96.50) | 0.288(vs.Gemini) 0.044*(vs.Llama) | 87.06 (84.31-89.81) | 0.490(vs.Gemini) <0.001*(vs.Llama) | 51.35 (39.96-62.74) | 13.64 (3.50-23.78) | 74 | 1.95 |
| SR4 (2025) | Gemini 2.0 | 90.00 (79.26-100.00) | 0.640(vs.Llama) 0.022*(vs.Qwen) | 98.27 (97.79-98.75) | <0.001*(vs.Llama) 0.006*(vs.Qwen) | 54.00 (40.19-67.81) | 10.00 (0.00-20.74) | 50 | 1.85 |
|  | Llama 3.1 | 93.33 (84.40-100.00) | 0.640(vs.Gemini) 0.006*(vs.Qwen) | 94.19 (93.34-95.04) | <0.001*(vs.Gemini) <0.001*(vs.Qwen) | 16.67 (11.03-22.31) | 6.67 (0.00-15.60) | 168 | 6.00 |
|  | Qwen 2.5 | 66.67 (49.80-83.54) | 0.022*(vs.Gemini) 0.006*(vs.Llama) | 99.10 (98.76-99.44) | 0.006*(vs.Gemini) <0.001*(vs.Llama) | 76.92 (60.72-93.12) | 33.33 (16.46-50.20) | 26 | 1.30 |
| SR5 (2026) | Gemini 2.0 | 100.00 (92.82-100.00) | 0.548(vs.Llama) 0.191(vs.Qwen) | 77.53 (73.25-81.81) | 0.169(vs.Llama) 0.273(vs.Qwen) | 82.93 (74.79-91.07) | 0.00 (0.00-7.18) | 82 | 1.21 |
|  | Llama 3.1 | 94.12 (88.53-99.71) | 0.548(vs.Gemini) 0.344(vs.Qwen) | 73.15 (68.60-77.70) | 0.169(vs.Gemini) 0.013*(vs.Qwen) | 65.31 (55.89-74.73) | 5.88 (0.29-11.47) | 98 | 1.53 |
|  | Qwen 2.5 | 89.71 (82.49-96.93) | 0.191(vs.Gemini) 0.344(vs.Llama) | 80.82 (76.78-84.86) | 0.273(vs.Gemini) 0.013*(vs.Llama) | 87.14 (79.30-94.98) | 10.29 (3.07-17.51) | 70 | 1.15 |

* Statistically significant difference

Note*: Wald 95% CIs are used by default. Wilson 95% CIs are used only for perfect evaluation scores, so boundary estimates retain finite-sample uncertainty.

Note**: Full-text evaluation metrics were calculated using model-specific full-text datasets generated from the outputs of title/abstract screening phase (i.e., TP, FP, and FN). Therefore, the number and composition of studies evaluated at the full-text phase differed across LLMs and review datasets.

Note**: Pairwise z-tests for Recall and %RW remain exploratory, unpaired comparisons of observed pipeline-level rates because LLMs received different full-text input cohorts; they are not matched head-to-head comparisons on identical articles.
