# Supplementary material for: Artificial intelligence-driven study selection in systematic reviews of randomized controlled trials, emulated trials and economic evaluation studies using large language models
Source: PLOS Digit Health. 2026 Aug 28;5(8):e0001668. doi: 10.1371/journal.pdig.0001668 (PMC13524291; doi:10.1371/journal.pdig.0001668)
Supplement: Supplementary file 14 [file pdig.0001668.s014.docx]

S14 Table Error analysis based on false negatives in the title and abstract screening phase and final classification in the full-text selection phase of each large language model

| **SR** | **LLM** | **Title/Abstract🡪Full-text** | **N** | **Domain** | **Reason for Exclusion: Title/Abstract** | **Reason for exclusion: Full-text** |
| --- | --- | --- | --- | --- | --- | --- |
| SR1 (2020) | Gemini 2.0 Flash | FN🡪FN | 0 |  |  |  |
|  |  | FN🡪TP | 0 |  |  |  |
|  |  | TP🡪FN | 0 |  |  |  |
|  | Llama 3.1 | FN🡪FN | 0 |  |  |  |
|  |  | FN🡪TP | 0 |  |  |  |
|  |  | TP🡪FN | 0 |  |  |  |
|  | Qwen 2.5 | FN🡪FN | 1 | P |  | Could not decide to exclude laparoscopic incision or secondary fascia closure (N = 1) |
|  |  |  |  | I&C | Could not identify mesh techniques (N = 1) | Could not identify mesh techniques (N = 1) |
|  |  |  |  | O | Could not decide whether outcomes occurred at the incision (N = 1) | Could not decide on hernia at non-incisional site as exclusion (N = 1) |
|  |  | FN🡪TP | 1 | I&C | Did not know that primary suture closure is same as no mesh closure (N = 1) |  |
|  |  | TP🡪FN | 3 | P |  | Could not decide to exclude laparoscopic incision or secondary fascia closure (N = 1) |
|  |  |  |  | I&C |  | Could not identify mesh techniques (N = 3) |
|  |  |  |  | O |  | Could not decide to exclude hernia at the non-incisional site (N = 1) |
| SR2 (2021) | Gemini 2.0 Flash | FN🡪FN | 1 | I&C | Could not identify intervention because only the title was available (N = 1) | Could not identify correct comparator (N = 1) |
|  |  | FN🡪TP | 1 | I&C | Did not know that deferred strategy is same as no prophylaxis (N = 1) |  |
|  |  | TP🡪FN | 1 | P |  | Could not identify patient from all organ transplants (N = 1) |
|  | Llama 3.1 | FN🡪FN | 0 |  |  |  |
|  |  | FN🡪TP | 0 |  |  |  |
|  |  | TP🡪FN | 0 |  |  |  |
|  | Qwen 2.5 | FN🡪FN | 2 | I&C | Could not identify intervention because only the title was available (N = 1) | Could not identify comparisons among active immunosuppressive regimens (N = 2) |
|  |  |  |  | O | Failed to select studies that did not have CMVs as outcome (N = 1) | Failed to select studies that did not have CMVs as outcome (N = 1) |
|  |  | FN🡪TP | 2 | I&C | Did not know that untreated and deferred strategies is same as no treatment (N = 2) |  |
|  |  | FN🡪NA | 1 | I&C | Could not identify intervention because only the title was available (N = 1) | Full text was not available for evaluation (N = 1) |
|  |  | TP🡪FN | 2 | I&C |  | Did not know that untreated and deferred strategies is same as no treatment, (N = 1) or historical control (N = 1) |
| SR3 (2024) | Gemini 2.0 Flash | FN🡪FN | 0 |  |  |  |
|  |  | FN🡪TP | 0 |  |  |  |
|  |  | TP🡪FN | 3 | P |  | Strictly included if all patients were 100% osteoporosis, not 50% or higher(N = 3) |
|  |  |  | 2 | O |  | Did not understand “any” outcome (N = 2) |
|  | Llama 3.1 | FN🡪FN | 0 |  |  |  |
|  |  | FN🡪TP | 0 |  |  |  |
|  |  | TP🡪FN | 1 | O |  | Did not understand “any” outcome (N = 1) |
|  | Qwen 2.5 | FN🡪FN | 5 | O | Did not understand “any” outcome (N = 5) | Did not understand “any” outcome (N = 5) |
|  |  | FN🡪TP | 1 | P | Did not know that prior fractures are different from primary osteoporosis (N = 1) |  |
|  |  |  | 2 | O | Did not understand “any” outcome (N = 2) |  |
|  |  | TP🡪FN | 1 | O |  | Did not understand “any” outcome (N = 1) |
| SR4 (2025) | Gemini 2.0 Flash | FN🡪FN | 0 |  |  |  |
|  |  | FN🡪TP | 0 |  |  |  |
|  |  | TP🡪FN | 5 | P |  | Could not understand age >=18 include all above age (N = 2) LLM classify mild ischemic stroke as TIA which is not a part of ASCVD (N = 1) It sticks to all subjects must be met eligible criteria, but the articles don’t (N = 2) |
|  | Llama 3.1 | FN🡪FN | 0 |  |  |  |
|  |  | FN🡪TP | 2 | O | Did not understand “any” outcome (N = 2) |  |
|  |  |  | 1 | S | Could not understand "stabilized inverse probability of treatment weighting" is one of emulated trials method (N = 1) |  |
|  |  | TP🡪FN | 1 | I&C |  | Could not understand that lipid-lowering therapy is a high-intensity stain (N=1) |
|  |  |  | 1 | S |  | Could not understand "stabilized inverse probability of treatment weighting" is one of emulated trials method (N = 1) |
|  | Qwen 2.5 | FN🡪FN | 2 | P | Could not understand criteria for identifying P (N = 2) (PCI and acute MI) | Could not understand criteria for identifying P (N = 2) (PCI and acute MI) |
|  |  |  | 1 | I&C | Could not understand "Low-Density Lipoprotein Cholesterol Target" is an eligible comparator. (N = 1) | Could not understand "Low-Density Lipoprotein Cholesterol Target" is an eligible comparator. (N = 1) |
|  |  | FN🡪TP | 3 | I&C | Could not know that the combination of drugs (ezetimibe) is eligible (N = 3) |  |
|  |  |  | 1 | S | Could not understand "propensity-score matching" is one of emulated trials method (N = 1) |  |
|  |  | TP🡪FN | 2 | P |  | It sticks to all subjects must be met eligible criteria, but the articles don’t (N = 1) Classified acute ischemic stroke as no ASCVD (N = 1) |
|  |  |  | 6 | I&C |  | Could not understand conventional dose is same as moderate dose (N=1) Did not understand “any” (N=4) It thinks too much that the comparators could not remained moderate intensity (N=1) |
| SR5 (2026) | Gemini 2.0 Flash | FN🡪FN | 0 |  |  |  |
|  |  | FN🡪TP | 0 |  |  |  |
|  |  | TP🡪FN | 0 |  |  |  |
|  | Llama 3.1 | FN🡪FN | 1 | I&C | Did not understand GDMT (N = 1) | Did not understand GDMT (N = 1) |
|  |  | FN🡪TP | 0 |  |  |  |
|  |  | TP🡪FN | 3 | I&C |  | Did not understand ARNI + SGLT2i is intervention. (N = 1) Did not understand term "any" (N=2) |
|  | Qwen 2.5 | FN🡪FN | 1 | I&C | Did not understand GDMT (N = 1) | Did not understand that comparator could be single medication (ACEi). it is looking for combination medicine. (N = 1) |
|  |  |  | 1 | S | Could not identify study design (N = 1) | model focus on the RCT not economic evaluation which is not follow our prompt instruction (N = 1) |
|  |  | FN🡪TP | 10 | I&C | model could not understand GDMT correctly (N = 10) |  |
|  |  | TP🡪FN | 3 | P |  | Could not understand term “any” type of HF (N = 3) |
|  |  |  | 2 | I&C |  | Could not understand term “any” (N = 2) |

FN, false negative; I&C, intervention & comparator; LLM, large language model; N, number; NA, full-text is not available; O, outcome; P, population; TP, true positive
