# Supplementary material for: Artificial intelligence-driven study selection in systematic reviews of randomized controlled trials, emulated trials and economic evaluation studies using large language models
Source: PLOS Digit Health. 2026 Aug 28;5(8):e0001668. doi: 10.1371/journal.pdig.0001668 (PMC13524291; doi:10.1371/journal.pdig.0001668)
Supplement: Supplementary file 15 [file pdig.0001668.s015.docx]

S15 Table The prediction results from the title and abstract screening to the full-text selection phases

| **SR** | **LLM** | **Prediction results from title/abstract screening to full-text selection phases** | | | | | | | |
| --- | --- | --- | --- | --- | --- | --- | --- | --- | --- |
|  |  | **Round** | **Test data** | **TP to TP** | **TP to FN** | **FP to FP** | **FP to TN** | **FN to FN** | **FN to TP** |
| SR1 (2020) | Gemini 2.0 Flash | 1 | 117 | 18 | 0 | 26 | 73 | 0 | 0 |
|  |  | 2 | 117 | 18 | 0 | 25 | 74 | 0 | 0 |
|  |  | 3 | 117 | 18 | 0 | 26 | 73 | 0 | 0 |
|  | Llama 3.1 | 1 | 114 | 18 | 0 | 84 | 12 | 0 | 0 |
|  |  | 2 | 114 | 18 | 0 | 84 | 12 | 0 | 0 |
|  |  | 3 | 114 | 18 | 0 | 84 | 12 | 0 | 0 |
|  | Qwen 2.5 | 1 | 36 | 13 | 3 | 8 | 10 | 1 | 1 |
|  |  | 2 | 36 | 13 | 3 | 8 | 10 | 1 | 1 |
|  |  | 3 | 36 | 13 | 3 | 8 | 10 | 1 | 1 |
| SR2 (2021) | Gemini 2.0 Flash | 1 | 316 | 18 | 1 | 46 | 249 | 1 | 1 |
|  |  | 2 | 316 | 18 | 1 | 45 | 250 | 1 | 1 |
|  |  | 3 | 316 | 18 | 1 | 46 | 249 | 1 | 1 |
|  | Llama 3.1 | 1 | 347 | 20 | 0 | 229 | 97 | 1 | 0 |
|  |  | 2 | 347 | 20 | 0 | 229 | 97 | 1 | 0 |
|  |  | 3 | 347 | 20 | 0 | 229 | 97 | 1 | 0 |
|  | Qwen 2.5 | 1 | 64 | 15 | 2 | 26 | 17 | 2 | 2 |
|  |  | 2 | 64 | 15 | 2 | 26 | 17 | 2 | 2 |
|  |  | 3 | 64 | 15 | 2 | 26 | 17 | 2 | 2 |
| SR3 (2024) | Gemini 2.0 Flash | 1 | 131 | 41 | 3 | 41 | 46 | 0 | 0 |
|  |  | 2 | 131 | 39 | 5 | 40 | 47 | 0 | 0 |
|  |  | 3 | 131 | 40 | 4 | 38 | 49 | 0 | 0 |
|  | Llama 3.1 | 1 | 160 | 43 | 1 | 75 | 41 | 0 | 0 |
|  |  | 2 | 160 | 43 | 1 | 75 | 41 | 0 | 0 |
|  |  | 3 | 160 | 43 | 1 | 75 | 41 | 0 | 0 |
|  | Qwen 2.5 | 1 | 110 | 35 | 1 | 36 | 30 | 5 | 3 |
|  |  | 2 | 110 | 35 | 1 | 36 | 30 | 5 | 3 |
|  |  | 3 | 110 | 35 | 1 | 36 | 30 | 5 | 3 |
| SR4 (2025) | Gemini 2.0 Flash | 1 | 135 | 26 | 4 | 22 | 83 | 0 | 0 |
|  |  | 2 | 135 | 25 | 5 | 22 | 83 | 0 | 0 |
|  |  | 3 | 135 | 27 | 3 | 23 | 82 | 0 | 0 |
|  | Llama 3.1 | 1 | 271 | 27 | 2 | 140 | 101 | 0 | 1 |
|  |  | 2 | 271 | 27 | 2 | 140 | 101 | 0 | 1 |
|  |  | 3 | 271 | 27 | 2 | 140 | 101 | 0 | 1 |
|  | Qwen 2.5 | 1 | 107 | 16 | 7 | 6 | 71 | 3 | 4 |
|  |  | 2 | 107 | 16 | 7 | 6 | 71 | 3 | 4 |
|  |  | 3 | 107 | 16 | 7 | 6 | 71 | 3 | 4 |
| SR5 (2026) | Gemini 2.0 Flash | 1 | 127 | 68 | 0 | 14 | 45 | 0 | 0 |
|  |  | 2 | 127 | 68 | 0 | 17 | 42 | 0 | 0 |
|  |  | 3 | 127 | 68 | 0 | 15 | 44 | 0 | 0 |
|  | Llama 3.1 | 1 | 140 | 64 | 3 | 34 | 38 | 1 | 0 |
|  |  | 2 | 140 | 64 | 3 | 34 | 38 | 1 | 0 |
|  |  | 3 | 140 | 64 | 3 | 34 | 38 | 1 | 0 |
|  | Qwen 2.5 | 1 | 99 | 51 | 5 | 9 | 22 | 2 | 10 |
|  |  | 2 | 99 | 51 | 5 | 9 | 22 | 2 | 10 |
|  |  | 3 | 99 | 51 | 5 | 9 | 22 | 2 | 10 |

FN, false negatives; FP, false positives; LLM, large language model; SR, systematic review project; TP, true positives; TN, true negatives
